# Epoxy composite dusts with and without carbon nanotubes cause similar pulmonary responses, but differences in liver histology in mice following pulmonary deposition

**DOI:** 10.1186/s12989-016-0148-2

**Published:** 2016-06-29

**Authors:** Anne Thoustrup Saber, Alicja Mortensen, Józef Szarek, Ismo Kalevi Koponen, Marcus Levin, Nicklas Raun Jacobsen, Maria Elena Pozzebon, Stefano Pozzi Mucelli, David George Rickerby, Kirsten Kling, Rambabu Atluri, Anne Mette Madsen, Petra Jackson, Zdenka Orabi Kyjovska, Ulla Vogel, Keld Alstrup Jensen, Håkan Wallin

**Affiliations:** 1 National Research Centre for the Working Environment, Lersø Parkallé 105, DK-2100 Copenhagen Ø, Denmark; 2 National Food Institute, Technical University of Denmark, Søborg, Denmark; 3 Faculty of Veterinary Medicine, University of Warmia and Mazury in Olsztyn, 10-719 Olsztyn, Poland; 4 Veneto Nanotech SCpA, ECSIN — European Centre for the Sustainable Impact of Nanotechnology, I-45100 Rovigo, Italy; 5 Queen’s University Belfast, University Road, Belfast, BT7 1NN Northern Ireland United Kingdom; 6 European Commission Joint Research Centre, Institute for Health and Consumer Protection, I-21027 Ispra, VA Italy; 7 Nanologica AB, SE-114 28 Stockholm, Sweden; 8 Department of Micro- and Nanotechnology, Technical University of Denmark, DK-2800 Kgs, Lyngby, Denmark; 9 Department of Public Health, University of Copenhagen, DK-1014 Copenhagen K, Denmark

**Keywords:** Nanoparticles, Nanomaterials, CNT, Nanocyl NC7000, Sanding dust, Epoxy, Matrix nanocomposite, Inflammation, DNA damage, Liver histology, Lifecycle

## Abstract

**Background:**

The toxicity of dusts from mechanical abrasion of multi-walled carbon nanotube (CNT) epoxy nanocomposites is unknown. We compared the toxic effects of dusts generated by sanding of epoxy composites with and without CNT. The used CNT type was included for comparison.

**Methods:**

Mice received a single intratracheal instillation of 18, 54 and 162 μg of CNT or 54, 162 and 486 μg of the sanding dust from epoxy composite with and without CNT. DNA damage in lung and liver, lung inflammation and liver histology were evaluated 1, 3 and 28 days after intratracheal instillation. Furthermore, the mRNA expression of *interleukin 6* and *heme oxygenase 1* was measured in the lungs and serum amyloid A1 in the liver. Printex 90 carbon black was included as a reference particle.

**Results:**

Pulmonary exposure to CNT and all dusts obtained by sanding epoxy composite boards resulted in recruitment of inflammatory cells into lung lumen: On day 1 after instillation these cells were primarily neutrophils but on day 3, eosinophils contributed significantly to the cell population. There were still increased numbers of neutrophils 28 days after intratracheal instillation of the highest dose of the epoxy dusts. Both CNT and epoxy dusts induced DNA damage in lung tissue up to 3 days after intratracheal instillation but not in liver tissue. There was no additive effect of adding CNT to epoxy resins for any of the pulmonary endpoints. In livers of mice instilled with CNT and epoxy dust with CNTs inflammatory and necrotic histological changes were observed, however, not in mice instilled with epoxy dust without CNT.

**Conclusions:**

Pulmonary deposition of epoxy dusts with and without CNT induced inflammation and DNA damage in lung tissue. There was no additive effect of adding CNT to epoxies for any of the pulmonary endpoints. However, hepatic inflammatory and necrotic histopathological changes were seen in mice instilled with sanding dust from CNT-containing epoxy but not in mice instilled with reference epoxy.

**Electronic supplementary material:**

The online version of this article (doi:10.1186/s12989-016-0148-2) contains supplementary material, which is available to authorized users.

## Background

Carbon nanotubes (CNTs) are very promising nanomaterials due to their many technically applicable properties. When CNTs are added to epoxy resins to form epoxy/CNT nanocomposites, these nanocomposites exhibit improved properties such as increased strength combined with reduced weight of the product [1, 2]. During the lifecycle of the nanocomposite (e.g., sanding, abrasion, shredding, incineration) CNTs may be released either as free particles or as part of a matrix.

Several studies on rodents have shown that pulmonary exposure to different types of CNTs induces an asbestos-like toxicological response characterized by persistent inflammation, granulomas and fibrosis with low no-effect levels [3–9]. It has been reported that abrasion particles from one type of epoxy/CNT composite is not cytotoxic in vitro [10] but little is known of the toxicity in vivo. The scientific literature on the toxicity of nanocomposites in general is very limited: to date, we are aware of five papers that have reported in vivo assessments of degradation fragments from other types of nanocomposites such as paints and lacquer with different nanoadditives [11–14], and plastic and cement with CNT [15]. In terms of inflammation, genotoxicity and histological lesions, none of these studies report increased toxicity of the sanding dust or other types of degradation fragments from nanocomposites compared to the products without nanomaterials.

Knowledge is currently developing on the process-specific particle emissions and release of fibrous nanomaterials during the life-cycle processes (e.g., sanding, weathering, shredding, and incineration) of carbon-based nanocomposites. Recently, it was shown that significant fractions of carbon fibers of μm-size diameters were clearly separated from matrix during industrial-scale grinding and sanding of layered silica-carbon epoxy composite [16]. Conversely, sanding of dispersed epoxy/CNT nanocomposite, using a smaller hand-held sander in laboratory setup produced only dust epoxy particles with protruding CNT [17]. The particle distributions were also found to be similar during sanding of epoxy/CNT nanocomposites and epoxy without CNTs. Similar observations has been made in other studies available [18–21].

The purpose of the present study was to assess the toxicity, by inflammatory and DNA damaging effect, of sanding dusts from epoxy composites with and without CNT for dose-responses following pulmonary exposure at different time points in mice. In order to be able to assess if the toxicological changes induced by dust from epoxy/CNT nanocomposites were similar to changes induced by the pristine CNT, data on the same CNT (Nanocyl NC7000) as used in the epoxy/CNT nanocomposite were included for comparison. Some of the data on the pristine CNT have been published previously [6, 22] and these were included for comparison. For the current study, we produced epoxy boards based on 1) an epoxy resin product with and without CNT for which we have full knowledge of content, and 2) an industrial epoxy resin Epocyl™ with the same CNT but with unknown mass content of CNT (<20 wt.%) and other additives. Epocyl™ is designed for industrial components, such as rollers, medical knifes and windmill blades, and for other applications in the following markets; automotive, sports, marine and aerospace [23]. For the toxicological testing, we chose to generate dust by sanding of the nanocomposites because this is a realistic life cycle scenario and allowed generation of a sufficient mass of collected dust for toxicity testing.

## Results

### Physicochemical characterization of particles and dusts

We tested sanding dusts from three different types of epoxy composite boards with and without CNT: The Danish Technological Institute provided two epoxy/CNT nanocomposite boards and one epoxy matrix board for the study: 1) One epoxy nanocomposite contained 0.2 % w/w CNT Nanocyl NC7000 (EPOXY-CNT), 2) One corresponding epoxy board contained no CNT (EPOXY-REF) and was considered a reference for the epoxy nanocomposite matrix; and 3) EPOCYL™NC R128-04 (EPOCYL) containing less than 20 wt.% Nanocyl NC7000 (material safety data sheet [24]). The pristine multi-walled Nanocyl NC7000 powder (denoted CNT) was included for comparison and carbon black Printex 90 (denoted CB) was included as reference material. Data regarding CNT NC7000 were published previously and will therefore not be described in detail [6].

#### Characteristics of the epoxyboards

Chemical characterisation by WDXRF was performed on disks cut out of the original boards. It was confirmed by SEM that these pieces (EPOXY-CNT and EPOCYL) contained CNT (Additional file 1: Figure S1). SEM images of polished EPOXY-CNT and EPOCYL reveal torn-off CNT fibers protruding the surface. The appearance of the composites was very similar. The clustering of the CNT indicates that CNT in the epoxy were not totally dispersed.

The elemental composition of the three epoxy materials was determined in solid disks (4 cm in diameter, 1 cm high) by wave-length dispersive X-ray fluorescence analysis (WDXRF). The results are shown in Additional file 2: Figure S2. For comparison, the results for the CNT Nanocyl powder, previously published in [25], were included in the figure. For better visualization, only the upper 0.3 % of the full axis is displayed. The three materials are chemically very similar; all are composed of 99.8 % C and between 0.12 and 0.16 wt.% Cl, plus 0.01 wt.% Si. All three contain Ni, Fe and Cu in trace amounts (<60 ppm). There was only a slight difference of less than 0.1 elemental weight% between EPOCYL and EPOXY-CNT can be observed. EPOCYL contained 0.07 wt.% Mo and 0.01 wt.% Mg, but no Mo or Mg was detected in the two other epoxy materials, or in NANOCYL or CB. Traces of S and P were detected in EPOCYL. The CNT-containing epoxy materials, EPOCYL and EPOXY-CNT, contained traces of Na, Al and Zn. Also CNT contained traces of Na, Al, Zn and other metal elements. CNT contributed with Fe and Al (>3 % in CNT) to the elemental composition of the CNT-containing epoxy.

#### Characteristics of the test materials used for the toxicological tests

**Fig. 1:**
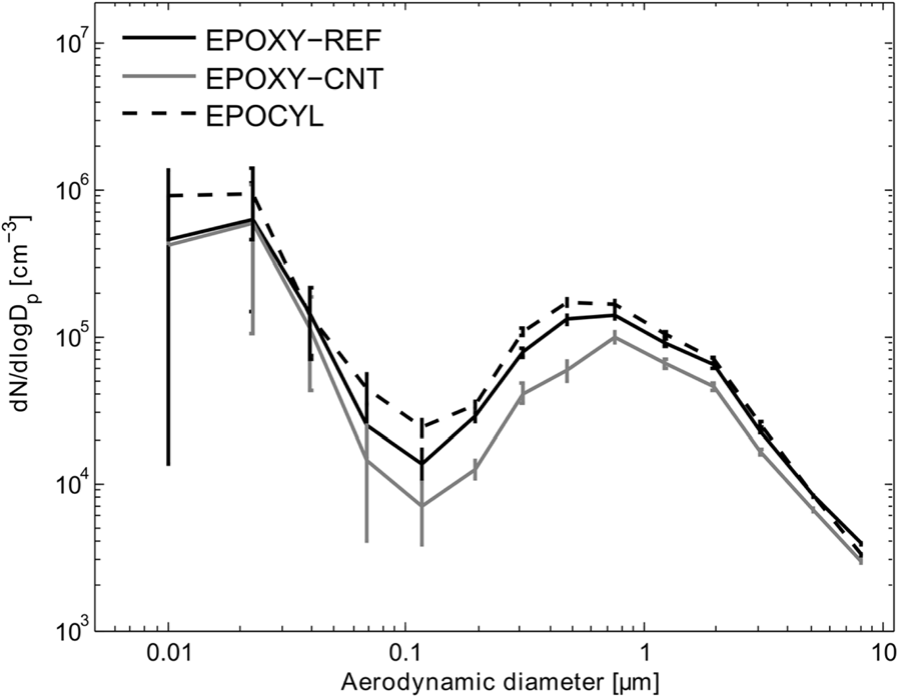
Average airborne dust particle size-distributions of the test materials measured using ELPI

Table 1 shows a summary of the key physicochemical characteristics of the test materials used in our study. The CB and CNT materials have been presented previously and we refer to these papers for more detailed descriptions [6, 26, 27]. The airborne sanding dusts were measured using an Electrical Low-Pressure Impactor (ELPI) and characterized morphologically by Scanning Electron Microscopy (SEM). The airborne particle ELPI number size-distributions peaked at approximately 20 nm and 700 nm. No apparent differences were observed between these size-modes of dusts generated by sanding of CNT-containing or CNT-free epoxy nanocomposites (Fig. 1). As we have reported before, the 20 nm size-mode is strongly dominated by particles generated by the electrical engine in the sanding machine [17, 28]. The 700 nm size-mode is also in good agreement with the size-distributions and size-resolved particle generation rates of the same plates studied in regular sanding tests [17].

**Table 1:** Overview of samples and average data on key physical chemical characteristics

Sample code	NRCWE ID number	Product form	Particle size^a^	BET specific surface area	Total pore volume	TGA mass loss	Main elemental impurities	ng endotoxin/mg particle^c^
CNT	NRCWE-026	Powder	11 ± 4 nm (diameter) 4+/− 0.4 μm (length)	246 m^2^/g	0.80 cm^3^/g	87 %	Al, Fe, Co^b^	BD
EPOXY-REF	NRCWE-034	Sanding dust	ND	4.53 m^2^/g	0.005 cm^3^/g	99 %	ND	3.43E-05
EPOXY-CNT	NRCWE-035	Sanding dust	ND	2.45 m^2^/g	0.003 cm^3^/g	99 %	ND	4.12E-06
EPOCYL	NRCWE-036	Sanding dust	ND	2.65 m^2^/g	0.003 cm^3^/g	99 %	ND	BD
CB		Powder	14 nm^d^	295−338 m^2^/g^e^	ND	ND	N (0.82 %), H(0.01 %)^e^	3.33E-03

*ND* not determined, *BD* below detection limit

^a^Average particle dimensions by SEM; ^b^Main elemental impurities as determined by ICP-MS [6]; ^c^Endotoxin content in particles/dust determined by *Limulus Amebocyte Lysate* test; ^d^According to manufacturer’s data; ^e^ [26] and [27]

**Fig. 2:**
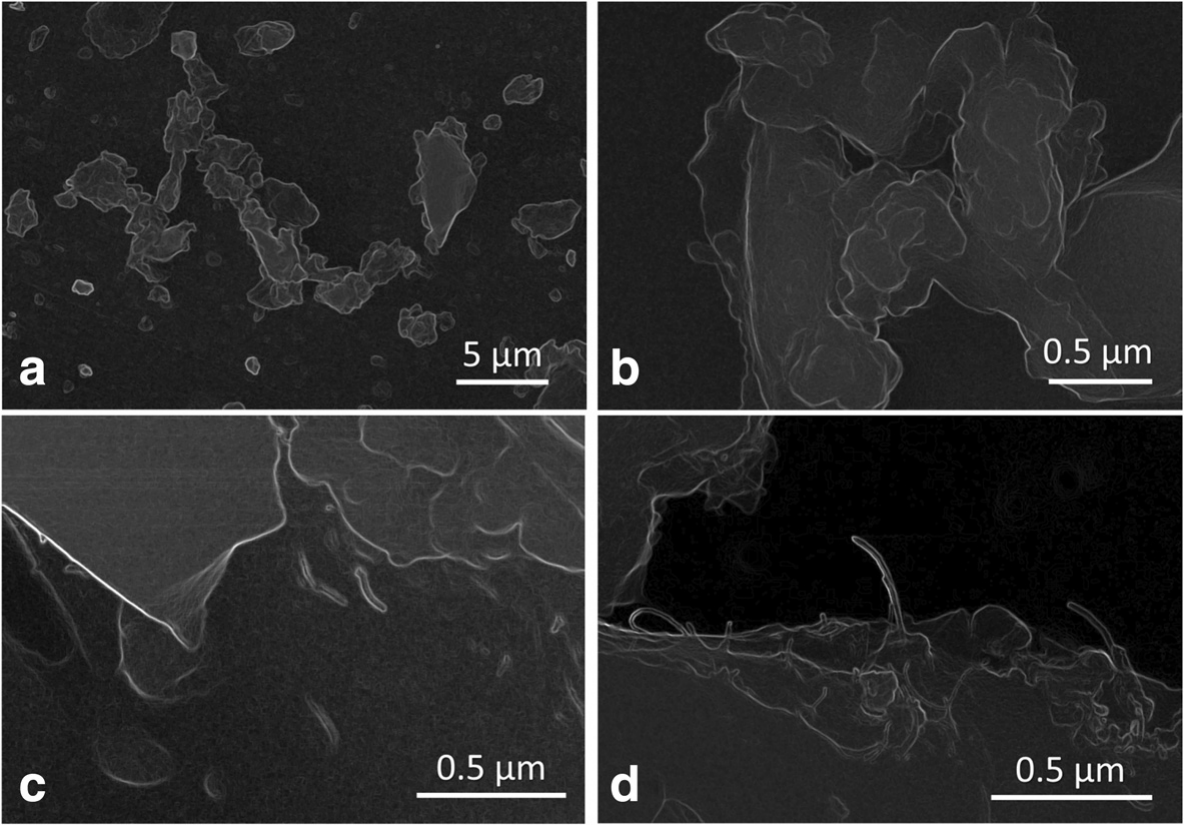
SEM-images of sanding dusts distributed onto two-size adhesive carbon tape. Examples of the particle surface morphology. **a** Overview showing the typical morphology and particle sizes of sanding dust from epoxy plates (EPOXY-REF), **b** Sanding dust from epoxy plates without CNT (EPOXY-REF), **c** Protruding CNT were rarely observed from BODOPOX added 0.2 % CNT (EPOXY-CNT), **d** Protruding CNT were frequently observed from sanding dust particles from EPOCYL added <20 % CNT (EPOCYL)

Scanning Electron Microscopy (SEM) of the test materials showed that the sanding dusts from epoxy/CNT nanocomposites were dominated by angular and sub-angular particles with upper sizes around 10 μm (Figs. 2a and b). Dusts generated by sanding of the EPOCYL and EPOXY-CNT had similar general morphology and sizes. CNT protruding from the surfaces were occasionally observed in dust sanding particles from EPOXY-CNT (Fig. 2c), but were abundant in dusts from EPOCYL (Fig. 2d). The protruding CNTs were clearly longer in NANOCYL than in EPOXY-CNT samples.

### Characterization of particles and dusts in instillation vehicle

#### Dynamic light scattering

The test materials were dispersed by probe-sonication in Nanopure-filtered water with 2 % v/v C57BL/6 mice serum and further diluted by serum-water into the concentrations used for instillation. All suspensions used for intratracheal instillation were analyzed by DLS (Dynamic Light Scattering). The DLS correlation plots suggested that the test materials dispersed well in the batch dispersions and instillation mediums, but the dispersions were often unstable as indicated by variable hydrodynamic sizes as well as trending hydrodynamic zeta-sizes and intensity counts (Table 2). The particle size-distributions of the sanding dust particles prevented full size-distributions of the raw dispersions used for instillation by DLS. However, acceptable size-distributions were observed for CB and CNT.

**Table 2:** Zeta-average (Z_ave_) and polydispersivity index (PDI) of the instillation mediums as measured by dynamic light-scattering

Dose	486 μg				162 μg				54 μg				18 μg			
Sample^c^	Z_ave_ (nm)	σ	PDI	σ	Z_ave_ (nm)	σ	PDI	σ	Z_ave_ (nm)	σ	PDI	σ	Z_ave_ (nm)	σ	PDI	σ
CNT–1					142	±6^a^	0.400	±0.019	119	±1	0.269	±0.008	111	±1	0.295	±0.024
CNT–2					139	±4^a^	0.389	±0.023	116	±2	0.369	±0.016	164	±6	0.471	±0.076
CNT–3													140	±2	0.415	±0.012
EPOXY-REF–1	^d^831	±97^b^	0.311	±0.037	^d^1212	±118^b^	0.424	±0.017	^d^831	±97^a^	0.281	±0.026				
EPOXY-REF–2					^d^858	±172^b^	0.228	±0.080								
EPOXY-CNT–1	^d^1197	±81^a^	0.464	±0.067	^d^1035	±50^b^	0.480	±0.026	^d^958	±113^a^	0.465	±0.045				
EPOXY-CNT-2					^d^1090	±104^b^	0.445	±0.029								
EPOCYL–1	^d^857	±98^b^	0.416	±0.042	^d^883	±71^b^	0.477	±0.033	^d^755	±111^b^	0.402	±0.049				
EPOCYL–2					^d^785	±59^b^	0.458	±0.054								
CB–1					87.5	±0.4	0.205	±0.008								
CB–2					90.1	±0.3	0.182	±0.008								

^a^The suspension was slightly unstable with less than 5 % change in derived count rate during measurement

^b^The suspension was highly unstable with more than 5 % change in derived count rate during measurement

^c^The numbers refer to different determinations of the same sample

^d^Upper size of particles in the sample exceeded the upper sizing-range of the DLS

#### SEM images of dust in instillation vehicle

The dispersibility of the test materials in the intratracheal instillation mediums was confirmed by scanning electron microscopy. The morphologies of the sanding dust particles were verified in the dispersion mediums where the protruding CNTs were also observed after sonication (see example in Fig. 2c).

#### Endotoxin

The endotoxin content in supernatants from particle suspensions used for intratracheal instillation was measured using the *Limulus Amebocyte* lysate enzyme assay (LAL) as previously described [12]. The amount of endotoxin received by mice given the highest tested dose (162 μg for CNT/CB and 486 μg sanding dust) was below 0.01 EU, a dose equivalent to 0.0005 ng endotoxin or 0.03 ng endotoxin/kg body weight.

### Cell count in broncho-alveolar lavage fluid

Mice received a single intratracheal instillation of 18, 54 and 162 μg of CNT or 54,162 and 486 μg of EPOXY-REF, EPOXY-CNT and EPOCYL. DNA damage in lung and liver, lung inflammation and liver histology were evaluated 1, 3 and 28 days after intratracheal instillation. Furthermore, the mRNA expression of *interleukin 6* (*Il6*) and *heme oxygenase 1* (*Hmox-1*) was measured in lung tissue and *Serum amyloid A1* (*Saa1*) was measured in liver tissue.

**Fig. 3:**
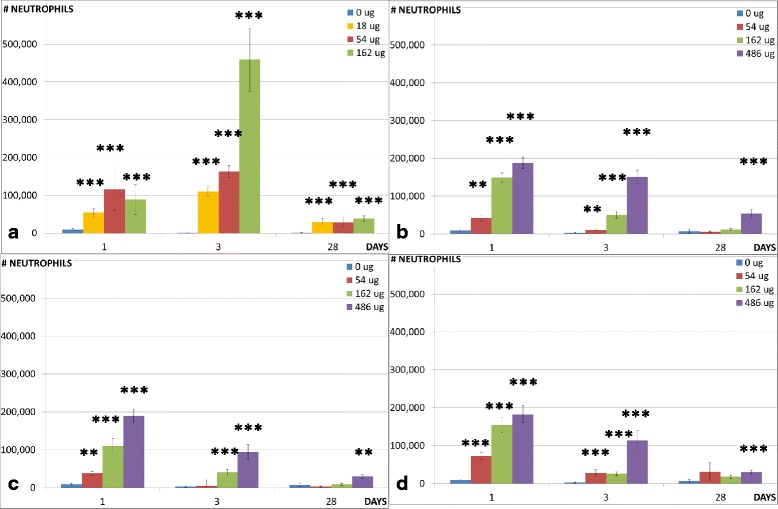
Neutrophil influx in the lungs. Neutrophil influx in the lungs of mice exposed to 0, 18, 54 or 162 μg of CNT (**a**) or 0, 54, 162 or 486 μg of EPOCYL (**b**), EPOXY-REF (**c**) or EPOXY-CNT (**d**). ^*^, ^**^, ^***^: Statistically significant compared to control mice at 0.5, 0.01 and 0.001 level, respectively. The CNT data has been published previously [6]

**Fig. 4:**
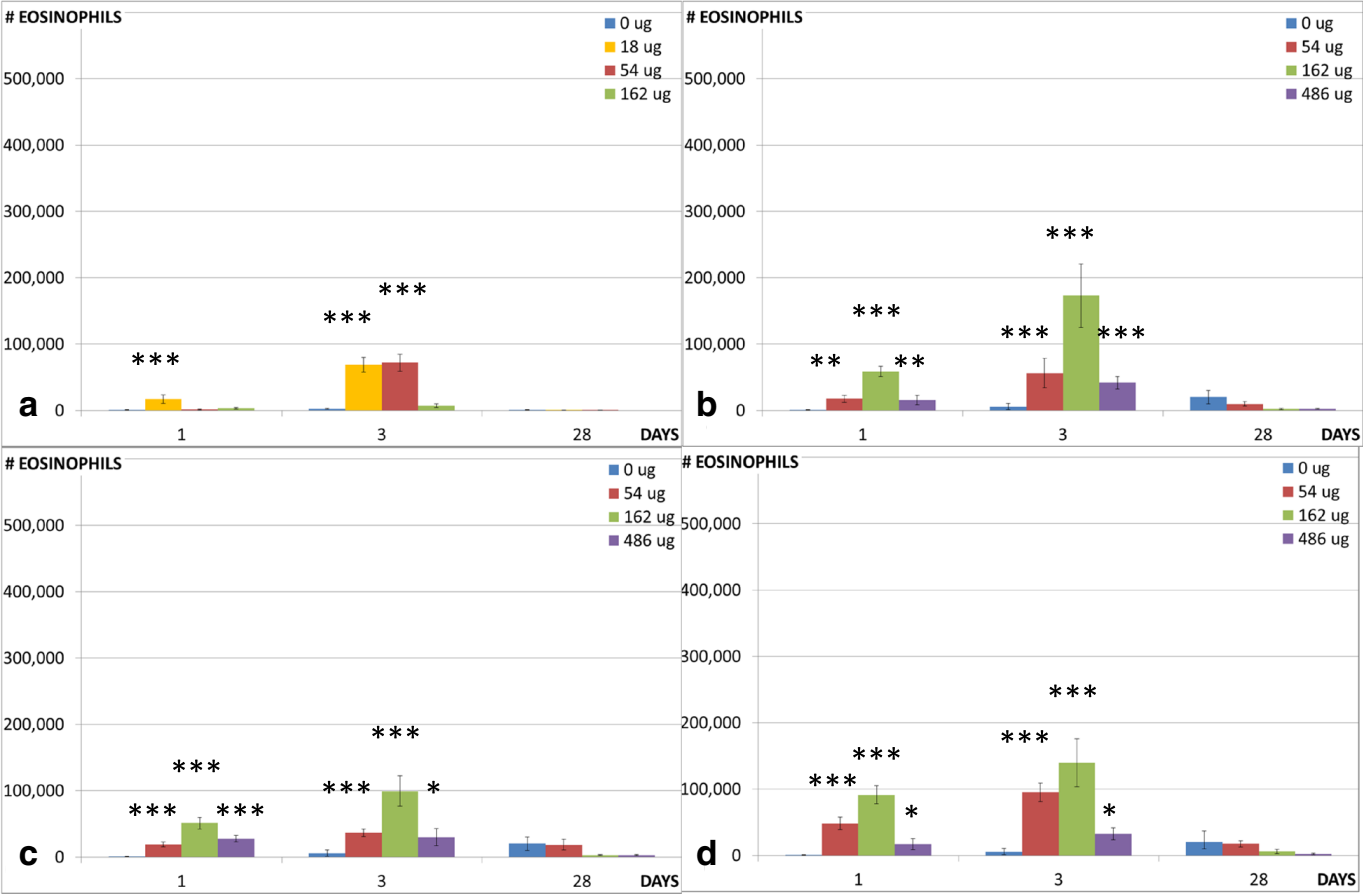
Eosinophil influx in the lungs. Eosinophil influx in the lungs of mice exposed to 0, 18, 54 or 162 μg of CNT (**a**) or 0, 54, 162 or 486 μg of EPOCYL (**b**), EPOXY-REF (**c**) or EPOXY-CNT (**d**). ^*^, ^**^, ^***^: Statistically significant compared to control mice at 0.5, 0.01 and 0.001 level, respectively. The CNT data has been published previously [6]

To assess the recruitment of inflammatory cells into the lung lumen, we determined the total number of BAL cells and the number of macrophages, neutrophils, eosinophils, lymphocytes, and epithelial cells in the BAL (Table 3). The neutrophil and the eosinophil influx are shown in Figs. 3 and 4, respectively. Previously published data on the CNT used in the epoxy are included for comparison [6].

**Table 3:** BAL fluid counts in mice 1, 3 and 28 days post exposure to 54 μg, 162 μg and 486 μg sanding dust from epoxy and 162 μg CB

		Control		EPOXY-REF			EPOXY-CNT			EPOCYL		CB^a^
1 day		0 μg	54 μg	162 μg	486 μg	54 μg	162 μg	486 μg	54 μg	162 μg	486 μg	162 μg
	Neutrophils (x10^3^)	9.4 ± 2.5	37.8 ± 5.0**	110.1 ± 20.8***	189.3 ± 16.2***	72.7 ± 8.8***	154.4 ± 20.1***	182.3 ± 22.2***	42.0 ± 7.8**	149.7 ± 12.1***	188.3 ± 15.2***	143.7 ± 26.1***
	Macrophages (x10^3^)	62.8 ± 5.9	93.5 ± 14.7	72.3 ± 16.7	49.3 ± 6.8	62.5 ± 5.1	70.6 ± 13.1	53.4 ± 2.9	85.8 ± 9.5	65.3 ± 11.6	41.4 ± 7.0	18.4 ± 4.5**
	Eosinophils (x10^3^)	1.0 ± 0.5	19.4 ± 3.7***	51.3 ± 8.7***	28.1 ± 5.0***	48.4 ± 9.4***	91.5 ± 13.7***	17.4 ± 8.5*	17.6 ± 5.3**	58.8 ± 7.9***	16.0 ± 7.0**	20.2 ± 5.7**
	Lymphocytes (x10^3^)	0.8 ± 0.2	2.8 ± 1.0	3.6 ± 1.0	4.6 ± 1.2*	5.2 ± 1.9	6.6 ± 2.7	6.8 ± 1.3*	2.1 ± 0.6	3.7 ± 1.3	3.5 ± 1.2	0.9 ± 0.5
	Epithelial (x10^3^)	8.8 ± 1.6	9.0 ± 1.7	10.7 ± 3.5	13.7 ± 4.2	9.7 ± 2.5	10.8 ± 3.1	13.1 ± 2.8	9.7 ± 2.2	10.6 ± 2.7	13.4 ± 1.7	9.8 ± 2.1
	Total BAL cells (x10^3^)	82.8 ± 8.3	162.4 ± 21.0**	248.0 ± 38.2***	285.0 ± 28.4***	198.5 ± 11.1***	334.0 ± 29.1***	273.0 ± 32.7***	157.2 ± 15.4**	288.0 ± 22.2***	262.5 ± 19.9***	193 ± 26.4**
3 days												
	Neutrophils (x10^3^)	2.6 ± 1.2	5.0 ± 1.3	40.9 ± 7.3***	94.5 ± 19.7***	28.0 ± 8.4***	26.2 ± 4.8***	113.5 ± 27.3***	10.5 ± 1.3**	50.6 ± 8.6***	151.5 ± 17.6***	120.2 ± 24.4***
	Macrophages (x10^3^)	57.8 ± 5.8	81.0 ± 12.5	96.9 ± 10.6	111.2 ± 22.2	78.0 ± 13.2	95.9 ± 16.4	125.1 ± 23.5*	84.8 ± 8.6	146.9 ± 11.8***	98.6 ± 32.4	65.5 ± 16.3
	Eosinophils (x10^3^)	5.3 ± 4.4	36.8 ± 5.8***	99.6 ± 22.8***	30.3 ± 12.9*	95.4 ± 14.0***	139.5 ± 36.1***	32.8 ± 9.0*	56.5 ± 22.1***	173.0 ± 47.7***	41.8 ± 9.5***	43.8 ± 16.3
	Lymphocytes (x10^3^)	1.7 ± 0.6	2.8 ± 0.6	29.6 ± 7.1***	53.7 ± 24.2***	10.6 ± 2.9*	34.2 ± 5.7***	35.5 ± 21.9	9.3 ± 3.5**	25.5 ± 5.4***	14.0 ± 3.5***	3.1 ± 1.4
	Epithelial (x10^3^)	7.1 ± 1.5	7.0 ± 0.7	9.5 ± 1.7	21.9 ± 2.4**	8.5 ± 2.6	10.3 ± 3.0	14.1 ± 3.4	11.9 ± 2.5	14.4 ± 2.6	24.6 ± 2.8**	12.8 ± 4.5
	Total BAL cells (x10^3^)	74.5 ± 8.8	132.5 ± 17.2	276.5 ± 34.3***	311.5 ± 64.4***	220.5 ± 33.4***	306.0 ± 46.1***	321.0 ± 67.9***	173.0 ± 30.8***	410.5 ± 52.0***	330.5 ± 31.6***	254.4 ± 28.3***
28 days												
	Neutrophils (x10^3^)	6.8 ± 4.2	3.4 ± 1.4	8.8 ± 3.2	29.6 ± 5.4**	6.9 ± 1.7	18.2 ± 4.1*	30.2 ± 5.0***	5.3 ± 2.1	12.0 ± 2.4	53.8 ± 9.8***	59.3 ± 8.6***
	Macrophages (x10^3^)	60.3 ± 5.7	53.1 ± 11.1	74.6 ± 8.4	100.2 ± 18.3	67.9 ± 12.7	93.1 ± 11.2	99.3 ± 11.9	50.9 ± 5.5	75.0 ± 5.9	84.2 ± 10.1	118.2 ± 29.5
	Eosinophils (x10^3^)	20.4 ± 10.1	18.5 ± 8.1	3.2 ± 0.7	3.2 ± 0.8	8.8 ± 2.7	6.3 ± 3.4	2.2 ± 1.0	10.0 ± 3.6	2.2 ± 0.9	2.5 ± 0.7	1.7 ± 1.0
	Lymphocytes (x10^3^)	4.9 ± 1.6	6.2 ± 2.1	5.7 ± 1.3	7.9 ± 1.6	4.7 ± 1.4	6.3 ± 0.8	10.1 ± 3.1	10.7 ± 4.0	6.4 ± 1.1	8.6 ± 1.3	36.4 ± 12.6
	Epithelial (x10^3^)	9.8 ± 1.9	11.8 ± 3.5	7.2 ± 2.6	13.5 ± 2.2	7.8 ± 2.6	7.6 ± 1.8	14.6 ± 2.7	10.1 ± 3.9	11.0 ± 2.1	12.9 ± 2.8	18.0 ± 3.0
	Total BAL cells (x10^3^)	102.3 ± 16.3	93.0 ± 19.2	99.5 ± 11.8	154.5 ± 18.1**	96.0 ± 17.0	131.5 ± 15.5	156.5 ± 12.8	87.0 ± 10.4	106.5 ± 7.5	162.0 ± 16.5*	233.5 ± 23.7**

Mean ± SEM

*p < 0.05 compared to controls, **p < 0.01 compared to controls, ***p < 0.001 compared to controls

There were no statistically significant differences between the three sanding dusts at the 0.05 level

^a^One mouse in the 162 μg CB group on day 3 post-exposure was considered an outlier because the total number of BAL cells was 10 times higher than the rest of the group. Therefore the BAL results from this mouse were excluded from the BAL dataset

Sanding dust from all epoxies resulted in increased numbers of total BAL cells in mice 1 day after intratracheal instillation of all doses (54, 162 and 486 μg). Significantly higher numbers of total BAL cells were also observed at all doses in mice 3 days after intratracheal instillation of the two CNT containing epoxies, while only 162 and 486 μg of the reference epoxy resulted in increased number of total BAL cells. Only the highest dose (486 μg) resulted in an increase in the total number of BAL cells 28 days after instillation of dust from the three epoxies.

Sanding dust from all epoxies resulted in increased neutrophil cell numbers in mice 1 day after intratracheal instillation of all doses (54, 162 and 486 μg). Significantly higher numbers of neutrophils were also observed at all doses in mice 3 days after intratracheal instillation of the two CNT containing epoxies, while only 162 and 486 μg of the reference epoxy resulted in increased number of neutrophils. Higher numbers of neutrophils were observed in mice 28 days after instillation of 486 μg of dust from EPOXY-REF, 162 and 486 μg of EPOXY-CNT and 486 μg of EPOCYL.

In mice instilled with sanding dust from epoxies, significantly higher number of macrophages were only seen 3 days after intratracheal instillation of EPOXY-CNT (486 μg) and EPOCYL (162 μg).

Sanding dust from all epoxies resulted in increased eosinophil counts in mice 1 and 3 days after intratracheal instillation of all doses (54, 162 and 486 μg). None of the tested materials resulted in increase in eosinophil counts 28 days after instillation.

One day after instillation, increased numbers of lymphocytes were only seen in mice instilled with the highest dose of EPOXY-REF and EPOXY-CNT (486 μg). Sanding dust from all epoxies resulted in increased lymphocyte counts 3 days after intratracheal instillation of the two highest doses (162 and 486 μg). In addition, instillation of 54 μg of dust from the two CNT containing epoxies resulted in increased number of lymphocytes. Only, instillation of EPOCYL resulted in increased lymphocyte numbers 28 days after instillation (54 and 486 μg).

Increased numbers of epithelial cells were only seen in mice 1 day after instillation of CNT and 3 days after instillation of 486 μg of two of the epoxy dusts (EPOXY-REF and EPOCYL).

Instillation of the reference particle (162 μg CB) resulted in a similar response as observed in our previous study [12, 29–31]: the inflammatory response was neutrophil dominated and persisted 28 days post-exposure. Increased total cell counts were observed at all time-points.

Thus, pulmonary inflammation was observed for all tested materials. There were no differences between mice intratracheally instilled with dust from the CNT containing epoxy (EPOXY-CNT) and the reference epoxy (EPOXY-REF) at any time point for any of the measured cell types, or between the two CNT containing epoxies (EPOXY-CNT and EPOCYL).

### *Il-6* and *Hmox-1* mRNA expression in the lungs

Sanding dust from all epoxy dusts resulted in increased *Il-6* mRNA expression level in lung tissue 1 day after intratracheal instillation of the two highest doses (162 and 486 μg) (Table 4). Significantly increased *Il-6* mRNA expression levels were also observed at the highest dose in mice 3 days after intratracheal instillation of EPOXY-REF and EPOCYL, while no increase of the dusts were seen on day 28 after exposure. There was no difference in *Il-6* mRNA expression levels between mice intratracheally instilled with dust from the CNT containing epoxy (EPOXY-CNT) and the reference epoxy (EPOXY-REF) at any time point, or between the two CNT containing epoxies (EPOXY-CNT and EPOCYL).

**Table 4:** Pulmonary mRNA expression levels in mice, 1, 3 and 28 days post-exposure to 54 μg, 162 μg and 486 μg sanding dust from epoxy and 162 μg CB

	Control	EPOXY-REF			EPOXY-CNT			EPOCYL			CB
1 day	0 μg	54 μg	162 μg	486 μg	54 μg	162 μg	486 μg	54 μg	162 μg	486 μg	162 μg
* Il-6*	0.08 ± 0.02	0.31 ± 0.19	1.19 ± 0.31***	2.33 ± 0.53***	1.45 ± 0.87**	2.32 ± 0.97***	1.91 ± 0.50***	0.26 ± 0.10*	2.43 ± 0.09***	1.35 ± 0.35***	0.21 ± 0.07
* Hmox-1*	5.26 ± 0.62	5.98 ± 1.5	13.8 ± 3.0	19.2 ± 2.7**	25.6 ± 10.2	28.9 ± 4.9**	17.2 ± 2.6*	6.85 ± 1.7	17.6 ± 3.8	15.6 ± 3.4	6.22 ± 0.89
3 days											
* Il-6*	0.07 ± 0.01	0.11 ± 0.03	0.51 ± 0.11**	0.6 ± 0.18***	0.11 ± 0.03	0.33 ± 0.15	0.90 ± 0.30	0.14 ± 0.07	0.25 ± 0.07	4.00 ± 1.28***	0.29 ± 0.11
* Hmox-1*	3.95 ± 0.72	4.07 ± 0.75	5.28 ± 1.3	6.47 ± 1.7	5.41 ± 1.5	9.11 ± 2.5	14.2 ± 5.7	7.32 ± 2.2	7.74 ± 2.6	28.3 ± 7.7	5.84 ± 1.45
28 days											
* Il-6*	0.24 ± 0.05	0.13 ± 0.03	0.21 ± 0.10	0.25 ± 0.08	0.15 ± 0.08	0.14 ± 0.05	0.17 ± 0.07	0.15 ± 0.03	0.11 ± 0.03	0.33 ± 0.11	0.21 ± 0.05
* Hmox-1*	4.95 ± 0.72	5.46 ± 1.3	9.33 ± 2.0	7.72 ± 1.3	3.40 ± 0.84	7.00 ± 2.3	7.38 ± 2.5	6.63 ± 1.0	5.70 ± 0.40	12.9 ± 1.04	4.87 ± 1.6

Normalised mRNA expression level of *Il-6* and *Hmox-1* (Mean ± SEM)

There were no statistically significant differences between the three sanding dusts at the 0.05 level

*p < 0.05 compared to controls, **p < 0.01 compared to controls, ***p < 0.001 compared to controls

Sanding dust from EPOXY-REF (the highest dose) and EPOXY-CNT (all doses) resulted in increased *Hmox-1* mRNA expression level in mice 1 day after intratracheal instillation, while no increase of the dusts were seen on day 3 and 28 after exposure (Table 4). There was no difference in *Il-6* mRNA expression level between mice intratracheally instilled with dust from the CNT containing epoxy (EPOXY-CNT) and the reference epoxy (EPOXY-REF) at any time point, or between the two CNT containing epoxies (EPOXY-CNT and EPOCYL).

### *Saa1* mRNA expression in the liver

**Fig. 5:**
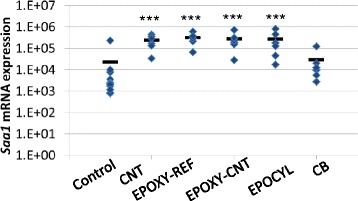
Hepatic *Saa1* mRNA expression. Normalised *Saa1* mRNA expression levels in the livers of mice exposed to 0 μg (control), 162 μg nanomaterial (CB or CNT) or 486 μg epoxy dust (EPOXY-REF, EPOXY-CNT or EPOCYL) 1 day after exposure. ^***^: Statistically significant compared to control mice at 0.001 level, respectively. There were no statistically significant differences between the three sanding dusts at the 0.05 level

Since we observed the highest pulmonary inflammatory response 1 day following intratracheal instillation we also measured *Saa1* mRNA expression in livers at the same time point from mice instilled with 486 μg of epoxy sanding dusts or 162 μg CB or CNT. Pulmonary exposure to CNT, EPOXY-REF, EPOXY-CNT and EPOCYL induced significant increases in the hepatic *Saa1* mRNA expression levels compared to controls, while there were no effects in mice exposed to CB (Fig. 5). There were no statistically significant differences in response between the three epoxy dusts or CNT.

### DNA damage

DNA damage was determined as DNA strand breaks and alkali labile sites in lung and liver tissue (Table 5) by the Comet assay.

**Table 5:** DNA damage (%T DNA) in lung and liver tissue, 1, 3 and 28 days post-exposure to 54 μg, 162 μg and 486 μg sanding dust from epoxy, 162 μg Printex 90 and control mice

	Control		EPOXY-REF			EPOXY-CNT			EPOCYL		CB
1 day	0 μg	54 μg	162 μg	486 μg	54 μg	162 μg	486 μg	54 μg	162 μg	486 μg	162 μg
Lung	3.25 ± 0.32	3.79 ± 0.30	4.95 ± 0.70*	4.89 ± 0.71*	4.37 ± 0.25	5.80 ± 0.74	4.78 ± 0.42	5.53 ± 0.38*	5.67 ± 0.43*	5.20 ± 0.46	4.60 ± 1.29
Liver	3.13 ± 0.47	3.90 ± 0.48	4.02 ± 0.55	3.30 ± 0.36	3.97 ± 0.71	3.08 ± 0.36	3.32 ± 0.48	3.47 ± 0.34	3.43 ± 0.23	3.07 ± 0.21	3.30 ± 0.24
3 days											
Lung	4.28 ± 0.35	4.33 ± 0.36	4.88 ± 0.81	4.45 ± 0.72	5.02 ± 0.74	3.89 ± 0.30	4.30 ± 0.45	4.78 ± 0.54	8.08 ± 1.19**	8.38 ± 2.15*	4.75 ± 0.61
Liver	3.29 ± 0.29	3.3 ± 0.36	3.22 ± 0.44	3.8 ± 0.38	3.25 ± 0.28	2.72 ± 0.24	3.52 ± 0.23	3.07 ± 0.21	3.4 ± 0.23	3.32 ± 0.24	3.62 ± 0.40
28 days											
Lung	4.71 ± 0.98	3.13 ± 0.31	4.28 ± 0.49	4.83 ± 0.25	3.70 ± 0.30	3.90 ± 0.45	5.72 ± 0.74	5.28 ± 0.93	4.40 ± 0.65	7.28 ± 1.92	4.67 ± 0.73
Liver	3.78 ± 0.34	3.37 ± 0.35	3.33 ± 0.17	2.78 ± 0.21	2.85 ± 0.27	3.38 ± 0.24	3.17 ± 0.25	3.43 ± 0.41	3.32 ± 0.36	3.63 ± 0.39	3.67 ± 0.55

Mean ± SEM

There were no statistically significant differences in response between the three epoxy dusts

*p < 0.05 compared to controls,**p < 0.01 compared to controls

#### Lung tissue

Pulmonary exposure to EPOXY-REF induced statistically significantly increased DNA strand break levels in lung tissue 1 day after intratracheal instillation of the two highest doses (162 and 486 μg), while there were no effects 3 and 28 days after instillation. EPOXY-CNT did not induce any significant increases in DNA strand break levels. In contrast, dust from EPOCYL induced significantly increase in DNA strand break levels 1 day after instillation of 54 and 162 μg, and 3 days after intratracheal instillation of 162 and 486 μg. There were no statistically significant differences in response between the three epoxy dusts. Previously published data on the CNT used in the epoxy showed that CNT induced pulmonary DNA damage on day 1 (18 and 54 μg) and day 3 (54 and 162 μg) [22].

#### Liver tissue

Compared to the vehicle controls, none of the test materials induced significantly changes in DNA strand break levels in liver tissue. There were no statistically significant differences between the three sanding dusts.

### Liver histology

**Fig. 6:**
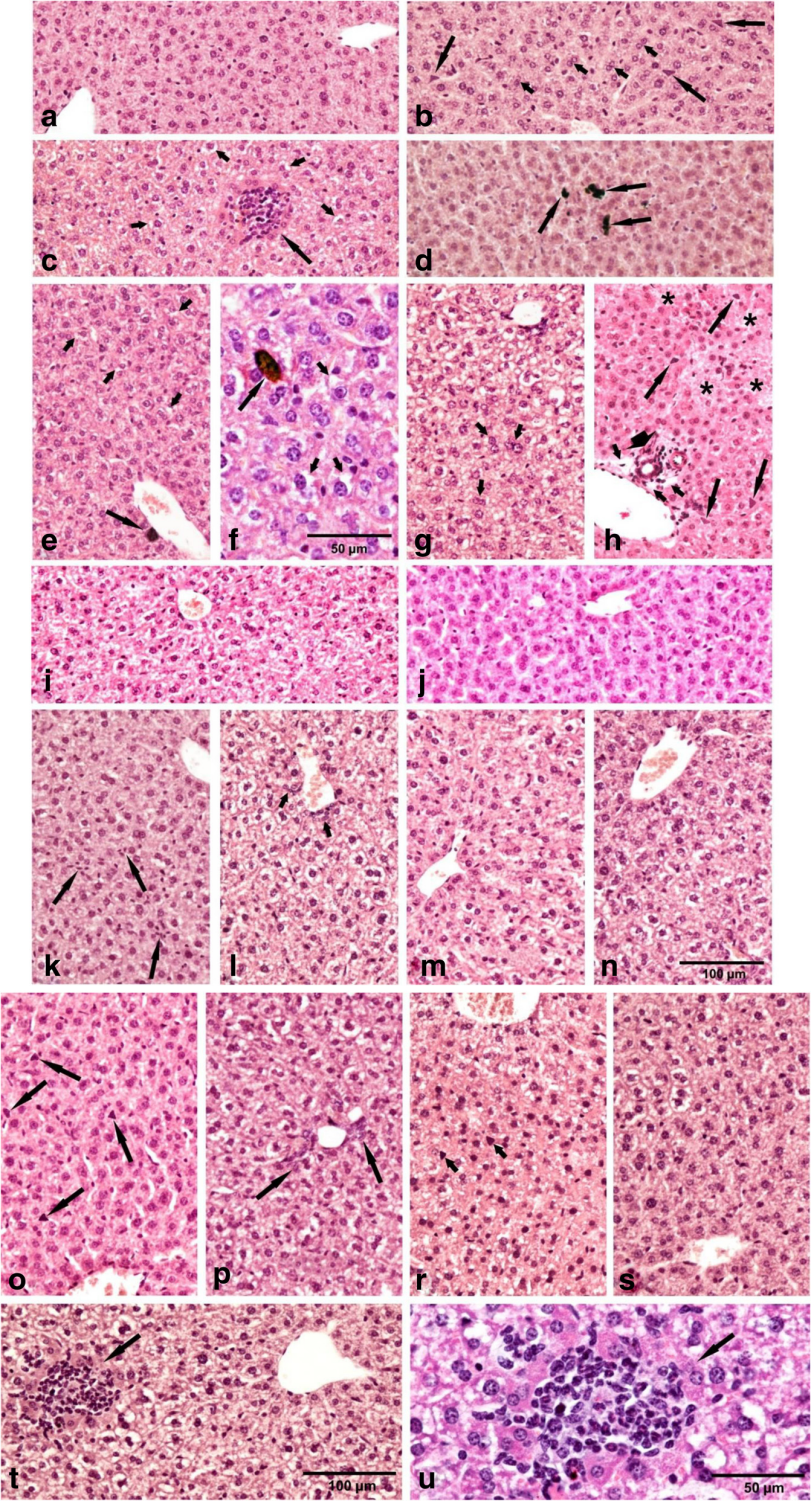
Histopathologic findings in the liver. **a**: typical microscopic pattern of the mouse liver – the control group. **b**-**h**: CNT exposed groups. **b**: 1 day after instillation (a.i.) to 54 μg: hypertrophy of Kupffer cells (*long arrows*), numerous binucleate hepatocytes (*short arrows*); **c**: 28 days a.i. to 54 μg: − granuloma surrounded by eosinophilic necrotic hepatocytes (*long arrow*), vacuolar degeneration (*short arrows*); **d**: 28 days a.i. to 54 μg: − macrophages (*arrows*), parenchymatous degeneration; **e**, **f**: 1 day after instillation (a.i.) to 162 μg: macrophages (*long arrows*), vacuolar degeneration (*short arrows*); **g**: 3 days a.i. to 162 μg: pronounced vacuolar degeneration in the central zone of the liver lobule, numerous binucleate hepatocytes (*arrows*); **h**: 28 days a.i. to 162 μg: foci of necrosis (*asterisks*), small granuloma (*head of arrow*), hypertrophy of Kupffer cells (*long arrows*), oedema (short arrows). **i**-**j**: The EPOXY-REF exposed groups, dose 486 μg **i** – 3 day a.i.: small degree vacuolar degeneration of hepatocytes; **j** – 28 days a.i.: typical pattern of the liver. **k**-**n**: The EPOXY-CNT exposed groups. **k** - 3 days a.i. to 162 μg: hyperplasia of Kupffer cells (arrows); **l** - 28 days a.i. to 162 μg: vacuolar degeneration on the whole area of the liver lobule, hyperplasia of the bile ducts epithelium (*arrows*); **m** - 28 days a.i. to 162 μg: mild-zonal vacuolar degeneration; **n** - 3 days a.i.to 486 μg: vacuolar degeneration of hepatocytes on the whole area of the liver lobule. Staining HE, magnification on the figures **a** – **e** and **g** - **m** as scale on the figure (**n). o**-**u: ** The EPOCYL exposed groups. **o** - 1 day a.i. to 54 μg: hypertrophy of Kupffer cells (*arrows*); **p** – 28 days a.i. to 54 μg: vacuolar degeneration on the whole area of the liver lobule, hyperplasia of the bile ducts epithelium (*arrows*); **r** - 1 day a.i. to 162 μg: vacuolar degeneration on the whole area of the liver lobule, hypertrophy of Kupffer cells (*arrows*); **s** – 3 days a.i. to 162 μg: mild-zonal vacuolar degeneration of hepatocytes; **t** - 3 days a.i. to 486 μg: vacuolar degeneration of hepatocytes on the whole area of the liver lobule, granuloma surrounded by eosinophilic necrotic hepatocytes (*arrow*), **u**: same pattern as on the figure (**t**) in double magnification. Staining HE, magnification on the figures (**o** – **s**) as scale on the figure (**t**)

Several histological changes were observed in the liver (Fig. 6, Table 6). For mice exposed to CNT, EPOXY-CNT and EPOCYL, the observed histological changes compared to controls were of the inflammatory, degenerative and necrotic types (Table 6). Histopathological findings in the liver have been reported previously for the CNT-exposed mice and these were included for comparison [22]. There was no difference in the type of lesions observed for mice exposed to CNT, EPOXY-CNT and EPOCYL and the incidences were comparable between the three groups. The granulomas in the mice instilled with the CNT or EPOCYL appeared bigger compared to granulomas in the livers of mice exposed to EPOXY-CNT. The degenerative changes in the three groups, observed one and three days after instillation were located in the central and middle zone of hepatic lobules. On day 28 after instillation, these lesions were mostly located peripherally regardless of the doses and the type of the test material with CNT.

**Table 6:** Type and incidence of histological lesions in the liver on days 1, 3 and 28 following exposure of mice to CNT, EPOXY-REF, EPOXY-CNT, EPOCYL or CB

Lesion	Control	CNT (μg/animal)			EPOXY-REF (μg/animal)			EPOXY-CNT (μg/animal)			EPOCYL (μg/animal)			CB (μg/animal)
	0	18	54	162	54	162	486	54	162	486	54	162	486	162
Foci (small) of inflammatory cells														
Day 1	0/12	0/6	0/5	0/6	0/6	0/6	0/6	0/6	0/6	0/6	0/6	0/6	0/6	0/6
Day 3	0/22	1/6	0/6	2/5	0/6	0/6	0/6	0/6	1/6	1/6	0/6	2/6	1/6	0/6
Day 28	0/24	2/6	1/6	1/6	0/6	0/6	0/6	1/6	2/6	2/6	1/6	2/6	2/6	0/6
Granuloma														
Day 1	0/12	0/6	0/5	0/6	0/6	0/6	0/6	0/6	0/6	0/6	0/6	0/6	0/6	0/6
Day 3	0/22	0/6	1/6	1/5	0/6	0/6	0/6	0/6	0/6	1/6	0/6	2/6	2/6	0/6
Day 28	0/24	2/6	2/6	1/6	0/6	0/6	0/6	0/6	1/6	1/6	1/6	1/6	2/6	0/6
Polymorphonuclear cell foci														
Day 1	0/12	0/6	0/5	0/6	0/6	0/6	0/6	0/6	0/6	0/6	0/6	0/6	0/6	0/6
Day 3	0/22	0/6	1/6	1/5	0/6	0/6	0/6	0/6	0/6	1/6	0/6	1/6	1/6	1/6
Day 28	0/24	2/6	1/6	3/6	0/6	0/6	0/6	1/6	1/6	0/6	1/6	1/6	2/6	2/6
Macrophages														
Day 1	0/12	2/6	1/5	1/6	0/6	0/6	0/6	1/6	0/6	0/6	0/6	1/6	0/6	0/6
Day 3	0/22	1/6	2/6	1/5	0/6	0/6	0/6	1/6	2/6	2/6	1/6	2/6	2/6	0/6
Day 28	0/24	1/6	2/6	2/6	0/6	0/6	0/6	0/6	2/6	2/6	1/6	1/6	1/6	0/6
Hyperplasia of connective tissue perivascular														
Day 1	0/12	0/6	0/5	0/6	0/6	0/6	0/6	0/6	0/6	0/6	0/6	0/6	0/6	0/6
Day 3	0/22	0/6	0/6	1/5	0/6	0/6	0/6	0/6	0/6	0/6	0/6	0/6	0/6	0/6
Day 28	0/24	0/6	0/6	1/6	0/6	0/6	0/6	0/6	0/6	0/6	0/6	0/6	1/6	0/6
Hyperplasia of connective tissue near bile ductless or venules														
Day 1	0/12	0/6	0/5	0/6	0/6	0/6	0/6	0/6	0/6	0/6	0/6	0/6	0/6	0/6
Day 3	0/22	0/6	0/6	1/5	0/5	0/6	0/6	0/6	0/6	0/6	0/6	0/6	0/6	1/6
Day 28	0/24	0/6	1/6	1/6	0/6	0/6	0/6	0/6	0/6	1/6	0/6	1/6	2/6	2/6
Microfoci of necrosis														
Day 1	0/12	1/6	0/5	0/6	0/6	0/6	0/6	0/6	0/6	0/6	0/6	0/6	0/6	0/6
Day 3	0/22	0/6	0/6	1/5	0/6	0/6	0/6	0/6	0/6	0/6	0/6	0/6	2/6	1/6
Day 28	0/24	1/6	2/6	2/6	0/6	0/6	0/6	0/6	0/6	1/6	0/6	1/6	1/6	1/6
Eosinophilic necrosis of single hepatocytes														
Day 1	0/12	0/6	0/5	0/6	0/6	0/6	0/6	0/6	0/6	0/6	0/6	0/6	0/6	0/6
Day 3	0/22	0/6	1/6	1/5	0/6	0/6	0/6	0/6	0/6	0/6	0/6	0/6	0/6	0/6
Day 28	0/24	2/6	1/6	2/6	0/6	0/6	0/6	1/6	0/6	1/6	0/6	1/6	1/6	1/6
Hepatocytes with pyknotic nuclei														
Day 1	0/12	0/6	0/5	0/6	0/6	0/6	0/6	0/6	0/6	0/6	0/6	0/6	0/6	0/6
Day 3	0/22	0/6	1/6	1/5	0/6	0/6	0/6	0/6	0/6	0/6	0/6	0/6	0/6	1/6
Day 28	0/24	1/6	2/6	2/6	0/6	0/6	0/6	1/6	0/6	1/6	1/6	1/6	2/6	1/6
Parenchymatous degeneration														
Day 1	0/12	0/6	0/5	0/6	0/6	0/6	0/6	0/6	0/6	0/6	0/6	0/6	0/6	0/6
Day 3	0/22	0/6	1/6	1/5	0/6	0/6	0/6	0/6	0/6	0/6	0/6	0/6	0/6	1/6
Day 28	0/24	1/6	2/6	2/6	0/6	1/6	0/6	0/6	1/6	1/6	0/6	2/6	2/6	2/6
Vacuolar degeneration														
Day 1	0/12	0/6	1/5	2/6	0/6	0/6	0/6	0/6	0/6	0/6	0/6	2/6	2/6	0/6
Day 3	0/22	2/6	2/6	3/5	0/6	0/6	1/6	0/6	1/6	2/6	1/6	2/6	3/6	2/6
Day 28	0/24	1/6	2/6	2/6	0/6	0/6	1/6	0/6	3/6	3/6	0/6	3/6	2/6	2/6
Binucleate hepatocytes														
Day 1	1/12	2/6	1/5	1/6	0/6	0/6	0/6	1/6	1/6	0/6	1/6	0/6	1/6	0/6
Day 3	1/22	1/6	2/6	2/5	1/6	2/6	1/6	0/6	1/6	2/6	1/6	2/6	1/6	2/6
Day 28	2/24	2/6	2/6	3/6	1/6	1/6	1/6	2/6	1/6	1/6	2/6	1/6	2/6	2/6
Oedematous endothelial cells of portal venules or close to blood vessels														
Day 1	0/12	0/6	0/5	0/6	0/6	0/6	0/6	0/6	0/6	0/6	0/6	0/6	0/6	0/6
Day 3	0/22	0/6	0/6	1/5	0/6	0/6	0/6	0/6	0/6	1/6	0/6	0/6	0/6	1/6
Day 28	0/24	0/6	2/6	2/6	0/6	0/6	0/6	0/6	1/6	1/6	0/6	1/6	2/6	2/6
Increased number (hyperplasia) of Kupffer cells														
Day 1	0/12	0/6	0/5	1/6	1/6	0/6	1/6	0/6	0/6	0/6	0/6	0/6	1/6	0/6
Day 3	0/22	1/6	1/6	2/5	0/6	0/6	0/6	0/6	2/6	2/6	1/6	1/6	1/6	1/6
Day 28	0/24	1/6	2/6	1/6	0/6	0/6	1/6	1/6	1/6	1/6	1/6	1/6	2/6	1/6
Hypertrophy of Kupffer cells														
Day 1	0/12	0/6	2/5	2/6	0/6	0/6	0/6	0/6	0/6	1/6	2/6	2/6	2/6	0/6
Day 3	0/22	0/6	3/6	2/5	0/6	1/6	1/6	1/6	2/6	1/6	1/6	2/6	1/6	0/6
Day 28	0/24	1/6	2/6	3/6	0/6	0/6	1/6	0/6	1/6	1/6	0/6	2/6	2/6	1/6
Hyperplasia of bile ducts epithelium														
Day 1	0/12	0/6	0/5	0/6	0/6	0/6	0/6	0/6	0/6	0/6	0/6	0/6	0/6	0/6
Day 3	0/22	1/6	2/6	1/5	0/6	0/6	0/6	0/6	0/6	0/6	1/6	0/6	1/6	0/6
Day 28	0/24	1/6	1/6	1/6	0/6	0/6	1/6	1/6	1/6	1/6	2/6	2/6	1/6	2/6

The histological changes in the livers from mice exposed to EPOXY-REF were similar to the controls with regard to the lack of the inflammatory and necrotic changes. For the other types of lesions recorded for the groups exposed to either CNT, EPOXY-CNT or EPOCYL low incidences were also noted in the EPOXY-REF group. Mice instilled with CB displayed hepatic changes of the type that we have reported before [12].

## Discussion

In the present study, we investigated the dose–response relationships of inflammation and DNA damage of respirable dust generated and sampled directly during sanding of epoxy boards with (EPOXY-CNT) and without CNT (EPOXY-REF) 1, 3 and 28 days after a single intratracheal instillation in mice. In addition, an industrial epoxy product with CNTs was included (EPOCYL). Our results show that pulmonary deposition of epoxy dust results in inflammation and pulmonary DNA damage up to 28 days and 3 days after exposure, respectively. There was no additive effect of adding CNTs to the epoxy compared to the reference epoxy for any of the measured pulmonary toxicological endpoints. In contrast, instillation with dusts from epoxy boards with CNT (EPOXY-CNT and EPOCYL) was associated with histological inflammatory and necrotic changes in the liver. These changes were also observed for CNT-instilled mice but not for mice instilled with dust from EPOXY-REF.

### Study design and dose considerations

We chose to study composite materials reinforced with the CNT Nanocyl NC 7000 because this CNT is widely used as reinforcement for many different applications including industrial components, such as rollers, medical knives and windmill blades, and for applications in the following markets; automotive, sports, marine and aerospace [2, 23]. The tested materials were chosen to represent a likely scenario of CNT-reinforced materials. In addition to EPOXY-REF and EPOXY-CNT for which we have full knowledge on content, we included a commercially available epoxy CNT composite (EPOCYL). The EPOXY-CNT contained 0.2 % CNT which was the largest amount of CNT that could be dispersed in the matrix. According to the safety datasheet, EPOCYL contained less than 20 % CNT. For this commercially available CNT-enforced epoxy composite we do not have a corresponding reference product and we do not have exact information on the contents.

The chosen doses (pristine nanomaterial:18, 54 and 162 μg, and sanding dust: 54, 162, 486 μg) and time points (1, 3 and 28 days) are similar to our previous study on sanding dusts from paints with and without nano titaniumdioxide (NanoTiO_2_) [12]. In that study, the tested nanopaint contained 10 % NanoTiO_2_ which made it possible to compare the toxicity of the NanoTiO_2_-containing paint dust to the toxicity of the same amount of both dust from paint without NanoTiO_2_ and to the toxicity of the same dose of pristine NanoTiO_2_ (eg. 18 μg of pristine NanoTiO_2_ corresponds approximately to the amount of NanoTiO_2_ in 162 μg of paint). A set-up enabling this comparison was not possible in the present study because of the low CNT content in EPOXY-CNT (0.2 %). Thus the 486 μg dose of EPOXY-CNT should be compared to the toxicity of less than1 μg of CNT and this dose was expected to be too low to generate a response. Higher doses than 486 μg of sanding dust was expected to result in overload. On the basis of these considerations and for comparison, we therefore chose to use the same doses of epoxy dusts as used in the previous study on paint dusts.

We have not been able to identify any studies on exposure levels to sanding dust in epoxy resin workers in the scientific literature. However, the dust doses (54, 162 and 486 μg) equal pulmonary deposition in mice after 8, 23 and 68 working days of 8 h at the Danish occupational exposure limit of 5 mg/m^3^ for respirable inert mineral dust, respectively (assuming that 10 % of the inhaled mass is deposited in the pulmonary region, volume of inhaled air per hour 1.8 l/h and 8 h working days). For comparison, the doses of CB (18, 54 and 162 μg) equal pulmonary deposition in mice after 1, 3 and 9 working days of 8 h at the Danish occupational exposure limit of 3.5 mg/m^3^ for CB, respectively, (with same assumptions as above except for a higher pulmonary deposition of CB in the pulmonary region (33 %)) [29]. When considering the recommended occupational exposure limit for CNTs of 1 μg carbon/m^3^ [5], the lowest dose of 18 μg/mouse corresponds to the calculated pulmonary deposition during a 40-year work life exposure assuming 10 % deposition [3], and a ventilation rate of 1.8 l/h.

### Pulmonary toxicity of epoxy composites

Mice intratracheally instilled with epoxy dust responded with a massive influx of polymorph nuclear cells into the lung lumen and the same response was observed for mice instilled with dust from EPOXY-CNT and EPOXY-REF. Two studies have been published on the pulmonary toxicity of dusts derived by machining of composite epoxy materials (graphite fiber-epoxy and fiberglass-epoxy) [32, 33]. Both reports are based on the same study of intratracheal instillation of the respirable fraction of six different types of composite epoxy dust (5 mg/200 g) in rats followed by an evaluation of endpoints 1 month after instillation. The neutrophil influx was 0.4 to 11.9 % of the BAL cells in the rats instilled with composite dust [33]. For comparison, in our study the similar dose (0.486 mg/20 g) resulted in a higher neutrophil influx (20–30 %) 28 days after exposure. Four of the six types of dust induced histopathological changes in the lungs that were more severe than aluminium oxide (negative control), while none of the dusts resulted in as severe changes as the ones that were observed in rats exposed to quartz (positive control) [32].

Mice intratracheally instilled with CNT and epoxy dust responded with a massive influx of eosinophils (previously discussed in [7]). Eosinophilia after CNT exposure has also been reported by others [34, 35]. Eosinophils have primarily been associated with allergic and asthmatic diseases [36]. Occupational exposure to epoxy resin hardener has been reported to cause eosinophilic bronchitis (reviewed by [37]).

### Hepatic effects of pulmonary deposition of epoxy/CNT composite dust

We observed a number of histological lesions (including granulomas) in the liver from mice exposed to EPOXY-CNT and EPOCYL dust compared to dust from EPOXY-REF. Compared to controls, none of the dusts caused increased levels of DNA strand breaks in liver tissue. Several histological liver changes, although of low incidence, were recorded in groups exposed to the dusts from either epoxy resin without CNT (EPOXY-REF) or from CNT containing epoxy resins (EPOXY-CNT and EPOCYL), as well as to CNT. The noteworthy finding was that inflammatory and necrotic changes were solely recorded in the CNT group and the groups exposed to the CNT-containing epoxy dusts. This indicates that the pulmonary deposition of these resins is associated with stronger hepatic effects compared to the pulmonary deposited epoxy without CNT (EPOXY-REF).

These hepatic changes could hypothetically be caused by systemic inflammation, other types of signalling and/or translocation of CNTs. The two latter possibilities are considered most likely, since the pulmonary inflammatory response was similar for EPOXY-CNT and EPOXY-REF and slow translocation of CNTs from the lungs to the liver and other distant organs has been reported [38, 39]. This is further supported by 1) the presence of dark material in macrophages in the livers from the CNT exposed mice suggesting translocation of CNTs, and 2) the presence of granulomas in the livers of mice exposed to EPOXY-CNT and EPOCYL which are similar to the granulomas detected in the CNT-exposed mice.

We have previously shown that intratracheal instillation of CNT (including NC7000) induced a strong pulmonary acute phase response in a dose-dependent manner [22]. The pulmonary acute phase response correlates closely with neutrophil influx [40]. The strong neutrophil influx observed for the epoxy dusts therefore indicate that pulmonary exposure to all epoxy dusts induces a pulmonary acute phase response. The pulmonary acute phase response following CNT exposure was accompanied by a hepatic acute phase response 1 and 3 days after exposure with *Saa1* as the most differentially regulated acute phase gene [22]. In addition, IL-6 is a known inducer of hepatic acute phase response [41] and was upregulated in mice exposed to the three epoxy dusts and CNT. We therefore assessed hepatic *Saa1* expression as biomarker of a hepatic acute phase response. Our results show that the hepatic mRNA expression levels of the acute phase gene *Saa1* were increased for all three sanding dusts and the CNT, while no increased *Saa1* mRNA expression levels were detected in the CB exposed mice (Fig. 5). During an LPS-induced hepatic acute phase response, several p450 genes including *Cyp1a2* are down-regulated [41]. In accordance with this, the mRNA expression levels of a number of Cyp450 genes including *Cyp7a1*, *Cyp3A44*, *Cyp1a2* were found to have lowered expression levels in liver from mice exposed to the CNT used in this study [22]. Furthermore, the time-course of lowered Cyp450 expression coincided with the acute phase response which was strongest on day 3. The increased hepatic *Saa1* expression following pulmonary exposure to the epoxy dusts thus indicates a hepatic acute phase response with an accompanying down-regulation of Cyp450 genes. However, since all epoxy dusts induced a pulmonary acute phase response, the acute phase response cannot explain the histological changes that were only observed in the livers from mice exposed to CNT-containing epoxy dust.

### Physico-chemical characteristics of importance for toxicity

No significant differences in the particle size distribution between EPOXY-REF and EPOXY-CNT was observed during sanding [17]. This is in agreement with previous findings from our study on sanding dusts from paints and lacquer with and without different additions of nanomaterials: a similar size distribution of dusts from similar products without nanomaterials was displayed [28]. A similar result was also published by Wohlleben and co-workers who studied the release of nanomaterials during abrasion of polyoxymethylene/CNT and cement/CNT nanocomposites [15]. Therefore, the difference in toxicity that we observed between the epoxy dusts cannot be explained by the particle size distributions.

Sanding epoxy boards generated two different size ranges below and above 100 nm. Similar size distributions have been observed in previous studies on particle generation during sanding of different types of materials by sanding machine engines [17, 28, 42]. The study by Gomez et al. was performed on the same epoxy materials and with the same sanding machine as in the present study. Gomez et al. showed that when the sanding machine was running on an empty load the number size distribution was dominated by particles between 10 and 50 nm [17]. We assume that the emissions by the sanding machine alone are dominated by Cu-rich particles [11]. The particles generated by the sanding machine alone may contribute to the observed toxicological effects. However, we do not believe that the hepatic changes in the present study can be explained by the smallest particles, because no histological changes in the liver were observed in our previous study of sanding dust from paints using the same sanding machine [11]. Thus, the sanding dust from paint contained the same 20 nm size-mode fraction, but induced no histological changes in liver. Therefore, we believe that the hepatic effects are not caused by the particles emitted by the sanding machine alone.

Sanding dusts from the epoxy/CNT composites were characterized by having single CNTs protruding from the surface of the nanocomposite particles. Protruding CNTs were also observed in the few other studies characterizing sanding dusts from epoxy/CNT composites [18–20]. The toxicological significance of sanding dust particles with these protruding CNTs is unknown. Hypothetically, a release of CNTs from the epoxy-matrix in the pulmonary region could result in translocation of CNTs to the systemic circulation and accumulation in the liver [43, 44]. As described above, the fact that we see dark material in macrophages in the livers from the CNT-exposed mice indicates that translocation of CNTs from the pulmonary region to the liver may have occurred. The presence of CNTs in the liver may contribute to the difference in hepatic toxicity that we observe.

We do not have information on the specific content of CNT in the industrial product, EPOCYL (stated to be <20 %) compared to the experimental CNT epoxy product. However, based on the very similar amounts of Fe and Al (the major trace elements in the used CNT) detected in the two types of CNT epoxy boards do not indicate that the content of CNT should be very different. Since we do not know the content of CNT in EPOCYL and we do not have a control sample without CNT for this epoxy, we cannot make any conclusions regarding the cause of the histological changes following exposure to EPOCYL dust.

It has been shown previously that the inflammatory response induced by low-toxicity low-solubility particles correlates well with the instilled surface area of the particles [12, 45–47]. In the current study, an association between deposited surface area and neutrophil influx was seen, but the association was no better than the corresponding association between neutrophil influx and instilled mass (Additional file 3: Figure S3). However, this study does not have an ideal design for assessing the effect of surface area.

We always include CB as an internal reference particle in our studies to be able to compare our results across studies. Moreover, the inclusion of CB in the study makes it also possible to compare the toxicity of spherical CB with fiber-shaped CNT. These two carbon based nanomaterials resulted in very different hepatic effects. Compared to CB, CNT induced much stronger hepatic *Saa1* mRNA expression level and some of the inflammatory lesions recorded for CNT were not observed following exposure to CB. Furthermore, our results also show that all three sanding dusts induced increased hepatic mRNA expression levels of the acute-phase gene *Saa1* while no increased *Saa1* mRNA expression levels were detected in the CB exposed mice. This suggests that the shape of nanomaterials with similar chemical composition is of importance for the toxicity. Shape has previously been shown to be a determinant of pulmonary response e.g., [48] but we do not know of reports of differences in the liver response following pulmonary exposure.

### Summary

Data on the toxicity of epoxy dust are scarce and to the best of our knowledge, no in vivo studies on the toxicity of particles generated during mechanical processing of epoxy/CNT nanocomposites have been published. However, in addition to the present study on epoxy/CNT composites, two other studies testing the same CNT (Nanocyl NC7000) as part of other matrices have been published by Wohlleben and co-workers: 1) testing of sanding dust from CNT/cement and CNT/polyoxymethylene matrices by pulmonary deposition in rats [15] and 2) in vitro test of sanding dust from CNT/polyurethane by “Precision Cut Lung Slices” [49]. This means that CNT Nanocyl NC7000 so far has been tested as additive in four different matrices and none of the studies have shown increased pulmonary toxicity of the CNT matrix compared to the reference matrix without CNT. However, of these only the present study has assessed hepatic effects.

## Conclusions

The level of pulmonary inflammation and DNA damage in mice pulmonary exposed to sanding dust from epoxy boards was not increased by the addition of CNT to the epoxy matrix. However, sanding dust from CNT-containing epoxy induced inflammatory and necrotic lesions in the liver that were not induced by EPOXY-REF but similar to the histological changes observed following pulmonary exposure to the same CNT.

## Methods

### Animals

Female C57BL/6 mice 5–7 weeks old (Taconic, Ry, Denmark) were acclimatized for 1–3 weeks before the experiment. Mice were given food (Altromin no. 1324, Christian Petersen, Denmark) and water ad libitum during the whole experiment. The mice were randomly divided into housing groups of 10in polypropylene cages (425 mm x 266 mm x 150 mm) with pinewood sawdust bedding and enrichment in form of sticks of aspen wood and rodent tunnels. The cages were stored at controlled temperature 21 ± 1 °C and humidity 50 ± 10 % with a 12-h light:12-h dark cycle.. Female mice were studied at 8 weeks of age. The average weight at the day of instillation was 18 ± 1.2 g. All animal procedures followed the guidelines for the care and handling of laboratory animals according to the EC Directive 86/609/EEC and the Danish law. The experiments were approved by the Danish “Animal Experiment Inspectorate” under the Danish Ministry of Justice (2012-15-2934-00223).

### Particles and sanding dusts

#### Products

The tested pristine nanomaterials comprised of a multiwalled CNT material (Nanocyl NC7000, CNT), and carbon black, Printex 90 (CB), which was included as an internal reference particle. Printex 90 was a gift from Degüssa (Germany). In addition, we tested sanding dusts from three different types of epoxy boards with and without CNT: The Danish Technological Institute provided three different types of epoxy boards: 1) an epoxy product with 0.2 % content of CNT (referred as EPOXY-CNT), 2) corresponding product without CNT (referred to as EPOXY-REF) and 3) EPOCYL (Table 1). EPOXY-CNT and EPOXY-REF were made of the epoxy resin BODOPOX 8000 (Bodotex, Vejle, Denmark) consisting of Bisphenol A and Bisphenol F. The CNTs were dispersed in the epoxy resin with 0 wt.% (EPOXY-REF) and 0.2 wt.% (EPOXY-CNT), respectively. For EPOCYL the used epoxy resin was an industrial product: EPOCYL™ NC RI 28–04 (Nanocyl S.A., Belgium). For all three products the curing agent was INF32 (Bodotex, Vejle, Denmark). The composites were cured at ca. 23 °C for ca. 24 h, followed by post-curing at 100 °C for minimum 24 h.

#### Characterization of epoxy boards

The inorganic chemical composition of the epoxy boards given as elemental weight % was measured by standardless wave-length dispersive X-ray fluorescence analysis (WDXRF) with a Tiger S8 4 kV instrument and SpectraPlus Vs.3 software (Bruker, Karlsruhe, Germany). The three epoxy materials were measured as solid disks (4 cm in diameter, 1 cm high).

Disks of 4 cm in diameter were cut out of the original material and polished using a polishing machine LaboPol (Struers, Ballerup, Denmark). Samples were imaged in a Helios EBS3 dual-beam SEM (FEI, Eindhoven, The Netherlands) at 2 kV, 86pA in in-lens mode.

### Generation of sanding dusts

**Fig. 7:**
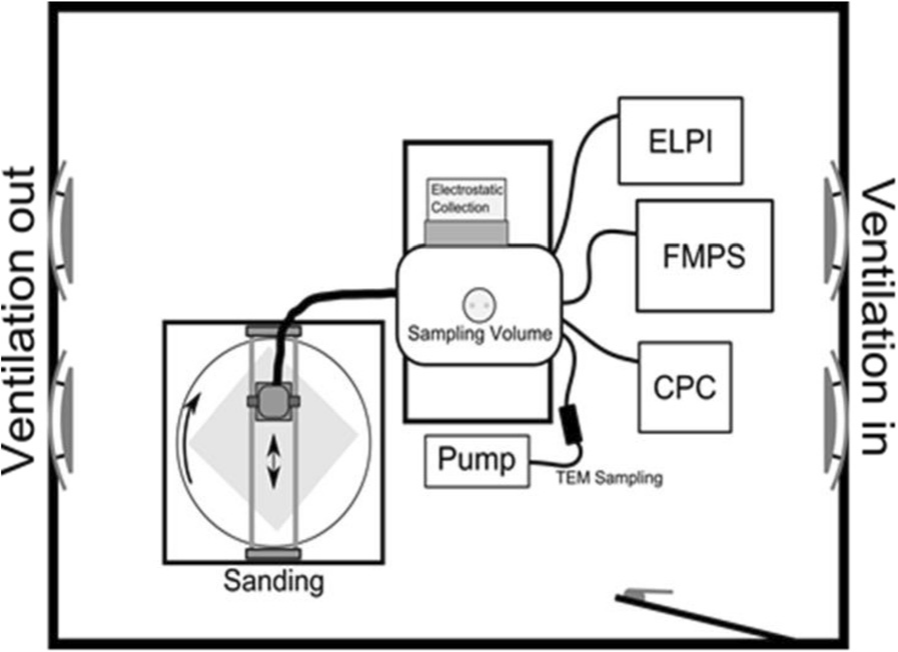
Experimental set-up. The dust is generated by sanding and collected by an electrostatic sampler

Figure 7 shows the experimental set-up used to perform sanding dust collection. It consists of a sander unit, a sampling tube, a 0.03 m^3^ dust reservoir made of plastic, and a commercial electrostatic precipitator (ESP), previously characterised by Sharma et al. [50], for particle sampling. In order to ensure a homogenous sanding of the surface, the plate material was mounted onto an electrically rotating disc, 5 rpm, and the sanding machine was locked in a uniaxial movement along the surface.

The size distribution of the generated particles was measured from the chamber using an ELPI+ (Electrical Low Pressure Impactor, Dekati Ltd., Finland), covering the range from 6 nm to 10 μm. The total particle concentration was measured using a CPC (Condensational Particle Counter, GRIMM). Sampling for analysis with electron microscopy was done with TEM-grids mounted in a 25 mm filter holder with a flow-through of 1 lpm.

Sanding was performed using a commercial hand-held orbital sander (Metabo Model FSR 200 Intec) with an internal fan for dust removal. Grit size 120 sanding paper was used as recommended by the paint and lacquer manufactures. For our purpose the sander outlet was modified to connect a 60 cm long and 32 mm ID flexible plastic tube after the sander to lead the dust to the dust reservoir chamber from which particle measurement and sampling was done.

The ESP was attached at the side of the chamber sampling air through a 21 cm deep, 37 cm wide and 15 cm high tunnel made in aluminium (Fig. 7). Sampling to the ELPI, CPC and TEM-grid was performed through 10 mm, TSI Conductive Silicone Tubing, connected to the end of the chamber.

The sanding procedure was initiated by a 1 min sampling of background air to ensure that a particle free environment was used. Thereafter the sander was initiated and ran for 30 s without touching the material surface. This was done to characterise the particle emissions from the sanding machine alone. The sanding machine was placed on the surface. Then the rotation of the plate and the back-forward motion of the sanding machine were started. The length of the sanding process varied from 5 min up to 30 min, depending on the amount of material created and collected by the ESP. After the ESP plates were saturated with dust, the sanding machine was turned off, the collected material were harvested and placed in glass jars.

#### Preparation of exposure stock

Particles were suspended by sonication in NanoPure water containing 2 % v/v serum collected from C57BL/6 mice. The serum was prepared from blood from unexposed mice yielding approximately 200 μ1 of serum per mouse. Serum was prepared by centrifugation of blood at 400 g (10 min, 4 °C). The particles (3.25 mg/ml) and dust suspensions (9.75 mg/ml) were sonicated using a Branson Sonifier S-450D (Branson Ultrasonics Corp., Danbury, CT, USA) equipped with a disruptor horn (Model number: 101-147-037) as described previously [12]. In brief, the sonication time was 16 min at 400 W and 10 % amplitude. These suspensions were used for the high dose (486 μg (dust) and 162 μg (CNT/Printex 90)) and diluted 1:3 for the medium dose and diluted further 1:3 for the low dose. Between the dilutions the suspensions were pipetted. Vehicle control solutions were prepared containing 98 % NanoPure water and 2 % serum.

### Particle and dust characterization

Nitrogen sorption isotherms were measured at liquid nitrogen temperature (77 K) using a Micromeritics ASAP2020 volumetric adsorption analyzer. Before the measurements, the samples were degassed under vacuum for 10 h at 80 °C. The BET (Brunauer- Emmett-Teller) equation [51] was used to calculate the surface area from adsorption data obtained in the relative pressure (p/po) range of 0.05 and 0.3. The total pore volume (Vtot) was calculated from the amount of gas adsorbed at p/po = 0.99. Pore size distribution curves were derived using Barrett-Joyner-Halenda (BJH) assuming a cylindrical pore model.

Thermogravimetric analyses were performed on Mettler TGA instrument by heating the samples from 25 to 900 °C at a heating rate of 5 °C min-1 on an alumina holder under the flow of air at 20 ml min-1.

The SEM analyses were performed by using a Zeiss NVision 40 Cross-Beam Focused Ion Beam machine, equipped with a high resolution Gemini Field Emission Gun (FEG) scanning electron microscope column. The instrument was also equipped with an Oxford INCA 350 Energy Dispersive X-Ray Spectrometer (EDS) incorporating an X-act silicon drift detector with an energy resolution of 129 eV at the Mn kα line.

A sample of the as-received carbon nanotubes was prepared by dispersing the powders directly on to a SEM stub covered with conductive carbon tape. SEM images were acquired at accelerating voltages of either 3 kV or 8 kV. EDS spectra were obtained from the samples at an accelerating voltage of 15 keV and a beam current 0.33 nA.

Samples of the sanding dusts were prepared on carbon tape on which a TEM grid was also mounted. The conductive tape was intended to facilitate SEM investigations but the dusts still exhibited highly non-conductive behaviour, which required careful adjustment of the working conditions. Accelerating voltages of 1–10 keV were used to minimize charging effects.

#### Characterization of dust and particle suspensions used for intratracheal instillation

##### Dynamic light scattering

The average sizes of the materials in instillation vehicle were determined by Malvern Nano ZS Dynamic Light Scattering (DLS) equipment mounted with a 633 nm red laser. The optical and dielectrical parameters of water were used for the medium while an optical refraction index of 2.5 and an optical absorption of 0.3 was used for the sanding dusts. The optical refraction index for CNT was set to 2.02 and the absorption was set to 2. Samples were thermally equilibrated to 25 °C in the DLS equipment before analysis. Each data point is the average of six consecutive analyses for each dispersion to measure the hydrodynamic size-distribution and the evolution of the derived count rate to assess the stability of the dispersions. The final data set was calculated considering the measured viscosity of the dispersion mediums using a SV-10 Vibro Viscometer (A&D, Japan)

##### Scanning electron microscopy

The dispersion state of the test materials in the instillation vehicle were characterized by scanning electron microscopy (SEM). Samples of CNTs in suspension (doses 18 μg and 162 μg) and dusts (doses 54 μg and 486 μg) used in the instillation experiments were prepared for SEM by depositing a small amount of the liquid on SEM-stubs covered with an Aluminum foil and allowing it to dry before examination. The SEM procedure was performed as described above.

##### Endotoxin

An amount of 3.24 mg/ml of each type of nanoparticle and 9.72 mg/ml of each type of dust was suspended in pyrogen free water with 0.05 % Tween 20 and suspended by sonication as described above. The endotoxin contents were analysed using the kinetic Limulus Amebocyte Lysate test (Kinetic-QCL endotoxin kit, Lonza, Walkersville, MD, USA) as described previously in [12].

#### Exposure of mice

The mice were treated with a single intratracheal instillation with 18, 54 and 162 μg for the nanoparticles and 54, 162 and 486 for the epoxy dusts (*n* = 5–7 per group). Because the CNT and sanding dust instilled mice were exposed in two overlapping experiments the number of control mice differs between the different time points: day 1 (12 mice), day 2 (22 mice) and on day 3 (24 mice). Before the intratracheal instillation, the mice were anesthetized using isoflurane. The instillation procedure has been described previously [12]. In brief, a 50 μl particle or dust suspension was instilled. Control animals were instilled with vehicle (2 % serum, 98 % NanoPure water).

#### Preparation of tissue and cells from the mice

One, 3 or 28 days after intratracheal instillation, tissue and cells were prepared as described previously [12]. In brief, following anesthesia with Hypnorm®/Dormicum®, a bronchoalveolar lavage (BAL) was performed by flushing the lungs twice using (1 ml/25 g body weight) saline in a 1 or 2 ml syringe. Each flush consisted of 3 up and down movements. The second flush was performed with fresh saline. The cellular composition of BAL cells was determined on 200 cells. The total number of cells was determined by using the NucleoCounter (Chemometec, Allerød, Denmark) live/dead assay according to the manufacturer’s instructions. The lungs and a piece of liver tissue were snap frozen in cryotubes (NUNC) in liquid N2 and stored at −80 °C. Another piece of liver tissue from the left lobe was kept in formaldehyde (4 %) until liver histology was performed.

#### RNA preparation from lung tissue and *Il-6* and *Hmox-1* real-time PCR

Total RNA was isolated from lung tissue of 144 mice in total (*n* = 6 mice per dose group) using the MagNA Pure Compact RNA Isolation kit (Roche) according to the manufacturer’s protocol. In brief, the RNA isolation procedure is based on the MagNA Pure Magnetic Glass Particle (MGP) Technology (Roche): nucleic acids are bound on the surfaces of MGPs whereas unbound molecules are removed by several washing steps. Genomic DNA molecules are degraded by incubation with DNase. Total RNA was stored at − 80 °C until analysis.

cDNA synthesis was performed using the Enhanced Avian HS RT-PCR kit (Sigma-Aldrich), with total RNA as template, as described in the manufacturer’s protocol. A total of 500 ng was used for each cDNA synthesis. The heating cycle was 25 °C (15 min)/50 °C (50 min)/85 °C (5 min) and the obtained cDNA solutions were further diluted to a final concentration of 10 ng/μl.

The expression of the target genes, compared to a reference (GAPDH), was determined with real time-PCR using a LightCycler® 480 Instrument (Roche) according to the manufacturer’s protocol. The relative expression was calculated using the Livak–Schmittgen method [52]. The statistical analyses were performed in Microsoft Excel through Mathematica (version 8, Wolfram Research). Statistical significance was calculated using a parametric one-way ANOVA.

#### RNA preparation from liver tissue and *Saa1* real-time PCR

RNA was prepared from liver tissue using using the Maxwell 16 LEV simplyRNA tissue kit as as described by the manufacturer (Promega Biotech AB, Sweden).cDNA synthesis was performed using the TaqMan® Reverse Transcription Reagents kit (ThermoFisher Scientific, Denmark), with total RNA as template, as described in the manufacturer’s protocol. The expression of hepatic serum amyloid A 1 (*Saa1*) and *18S* was measured using a modified TaqMan Fast 2x Universal PCR Master Mix protocol (ThermoFisher Scientific, Denmark). The primer/probe mix for *Saa1* was Mm00656927_g1 (ThermoFisher Scientific, Denmark) and *Saa1 *mRNA levels were normalised to* 18S*. The samples were run in triplicates on 384-well reaction plates (Thermo Fisher Scientific, Denmark). A negative (minus reverse transcriptase), a positive and a blank control were added on each plate. The plate was run in the ViiA 7 Real-time PCR system (Thermo Fisher Scientific, Denmark). The relative expression was calculated using the Livak–Schmittgen method [52].

#### Comet assay

The level of DNA strand breaks in frozen lung and liver tissue was determined by the alkaline comet assay using Imstar as described previously in [53]. As a positive assay control and to estimate the electrophoresis-to-electrophoresis variation, 0 and 30 μM H_2_O_2_ exposed A549 cells were included on each Gelbond film in all electrophoresis runs.

#### Liver histology

Specimens were taken from the liver of five to six mice from the vehicle control from the particle and dust dose groups of all test materials 1, 3 or 28 days after instillation. The specimens were fixed in 4 % neutral buffered formaldehyde, paraffin-embedded, and sections 4–6 μm were made and stained with hematoxylin and eosin for histological examination under the Nikon Eclipse 80i optical microscope equipped with a Nikon PS – Fi1 digital camera (Eclipse 80i; Nikon, Japan) using NIS-Elements BR 2.30 programme (Nikon, Japan).

#### Statistics

For each particle, the data were assessed by non-parametric two-way ANOVA with post-hoc Tukey-type multiple comparison test for effects showing statistical significance in the overall ANOVA test. A comparison of the three dusts was assessed by non-parametric three-way ANOVA. Statistical significances were tested at *P* < 0.05 level. The statistical analyses were performed in SAS version 9.2 (SAS Institute Inc., Cary, NC, USA).

## Abbreviations

BAL, bronchoalveolar lavage; BET, Brunauer-Emmett-Teller; BJH, Barrett-Jouner-Halenda; CB, carbon black (Printex 90); CNT, carbon nanotubes; CPC, condensation particle counter; DLS, dynamic light scattering; EDS, energy dispersive x-ray spectrometer; ELPI, ELECTRICAL LOW-PRESSURE IMPACTOR; EPOCYL, industrial epoxy composite with CNT; EPOXY-CNT, epoxy composite with CNT; EPOXY-REF, epoxy composite without CNT; ESP, ElectroStatic Precipitator; FEG, field emission gun; Hmox-1, heme oxygenase; IL-6, interleukin 6; LAL, limulus amebocyte lysate; NanoTiO_2_, nano titaniumdioxide; Saa1, serum amyloid a1; SEM, scanning electron microscopy; Vtot, total pore volume; WDXRF, wave-length dispersive x-ray fluorescence

## Additional files

Additional file 1: Figure S1. SEM images of A) EPOXY-CNT and B) EPOCYL polished surfaces with CNT (torn-off ends) sticking out. The materials are very similar in appearance. (PPTX 713 kb)

Additional file 2: Figure S2. Inorganic chemical composition given as elemental weight% measured by standardless WDXRF. The three epoxy materials were measured as solid disks (4 cm in diameter, 1 cm high). For comparison, the results for CNT powder, previously published in [25], were added to the figure. Displayed axis 99.7 – 100 %. (PPTX 71 kb)

Additional file 3: Figure S3. Correlation between neutrophil influx and mass (A) or surface area (B) of the instilled particles and sanding dusts. (PPTX 122 kb)
