# Supplementary figures and images for: Epoxy composite dusts with and without carbon nanotubes cause similar pulmonary responses, but differences in liver histology in mice following pulmonary deposition
Source: Part Fibre Toxicol. 2016 Jun 29;13:37. doi: 10.1186/s12989-016-0148-2 (PMC4928277; doi:10.1186/s12989-016-0148-2)

## Slide 1
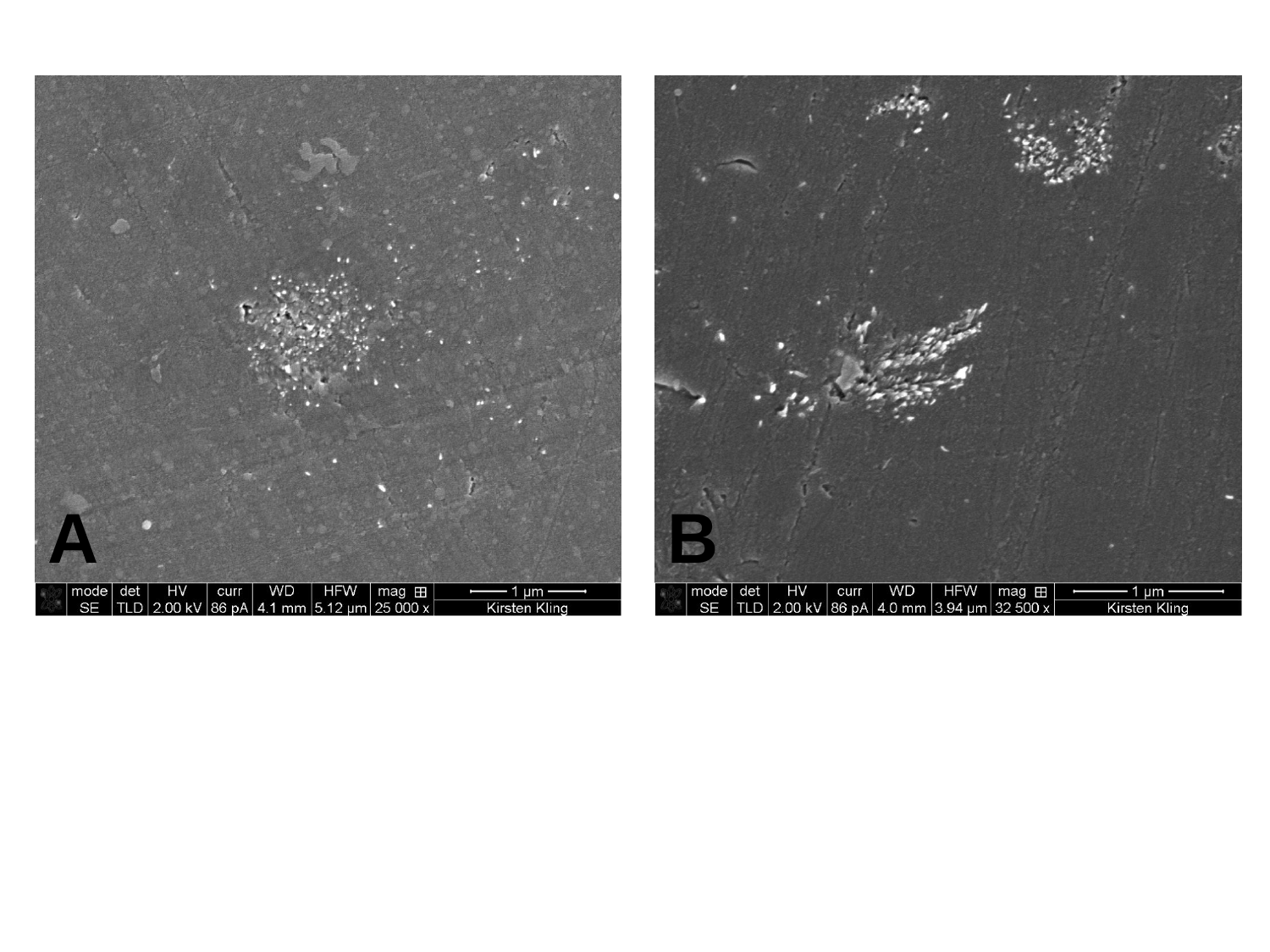

A
B

Supplement: Supplementary file 1 — SEM images of A) EPOXY-CNT and B) EPOCYL polished surfaces with CNT (torn-off ends) sticking out. The materials are very similar in appearance. (PPTX 713 kb) [file 12989_2016_148_MOESM1_ESM.pptx]

## Slide 1
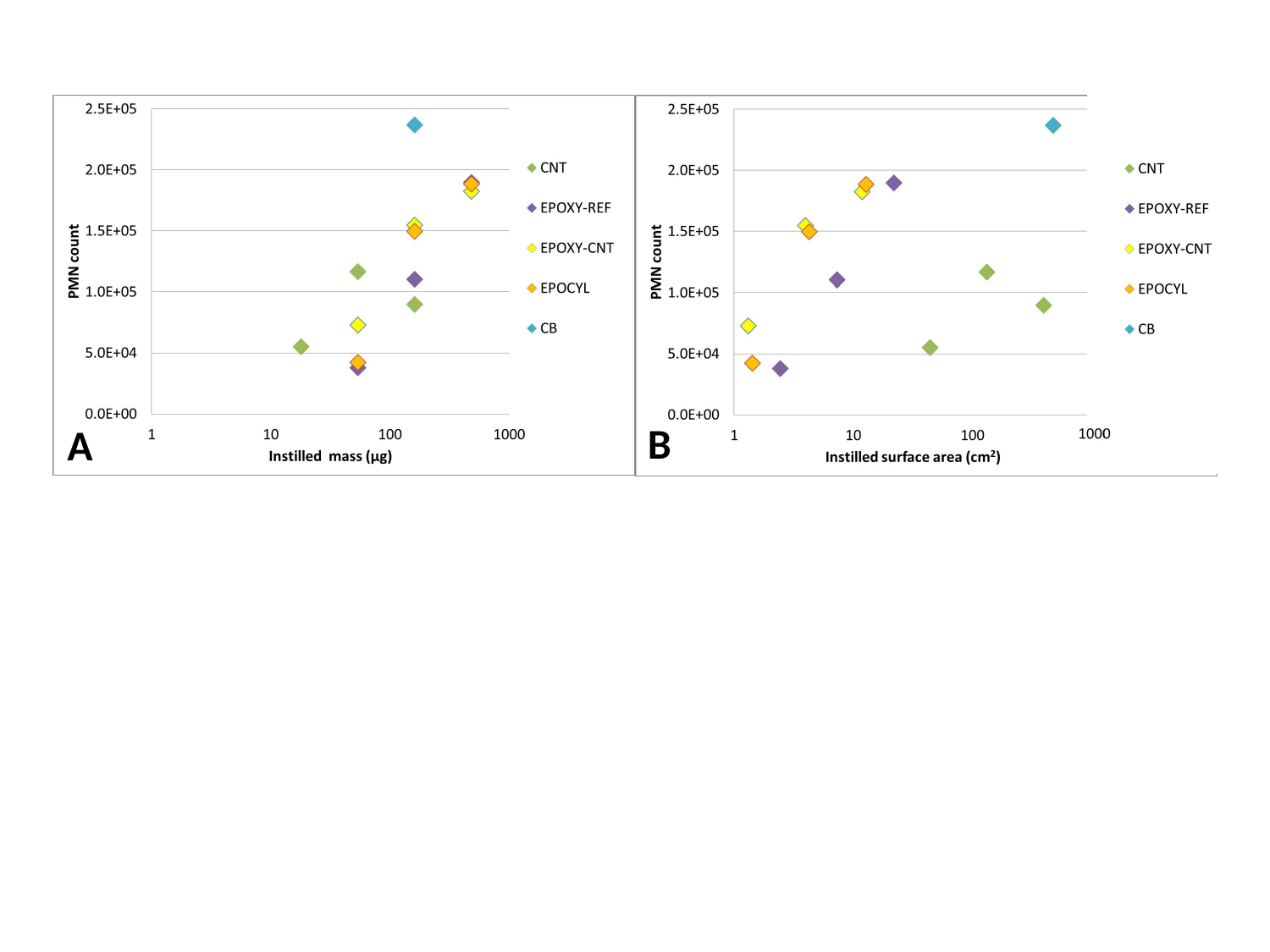

B
A

Supplement: Supplementary file 3 — Correlation between neutrophil influx and mass (A) or surface area (B) of the instilled particles and sanding dusts. (PPTX 122 kb) [file 12989_2016_148_MOESM3_ESM.pptx]
