# Supplementary material for: Epoxy composite dusts with and without carbon nanotubes cause similar pulmonary responses, but differences in liver histology in mice following pulmonary deposition
Source: Part Fibre Toxicol. 2016 Jun 29;13:37. doi: 10.1186/s12989-016-0148-2 (PMC4928277; doi:10.1186/s12989-016-0148-2)
Supplement: Supplementary file 2 — Inorganic chemical composition given as elemental weight% measured by standardless WDXRF. The three epoxy materials were measured as solid disks (4 cm in diameter, 1 cm high). For comparison, the results for CNT powder, previously published in [25], were added to the figure. Displayed axis 99.7 – 100 %. (PPTX 71 kb) [file 12989_2016_148_MOESM2_ESM.pptx]

## Slide 1
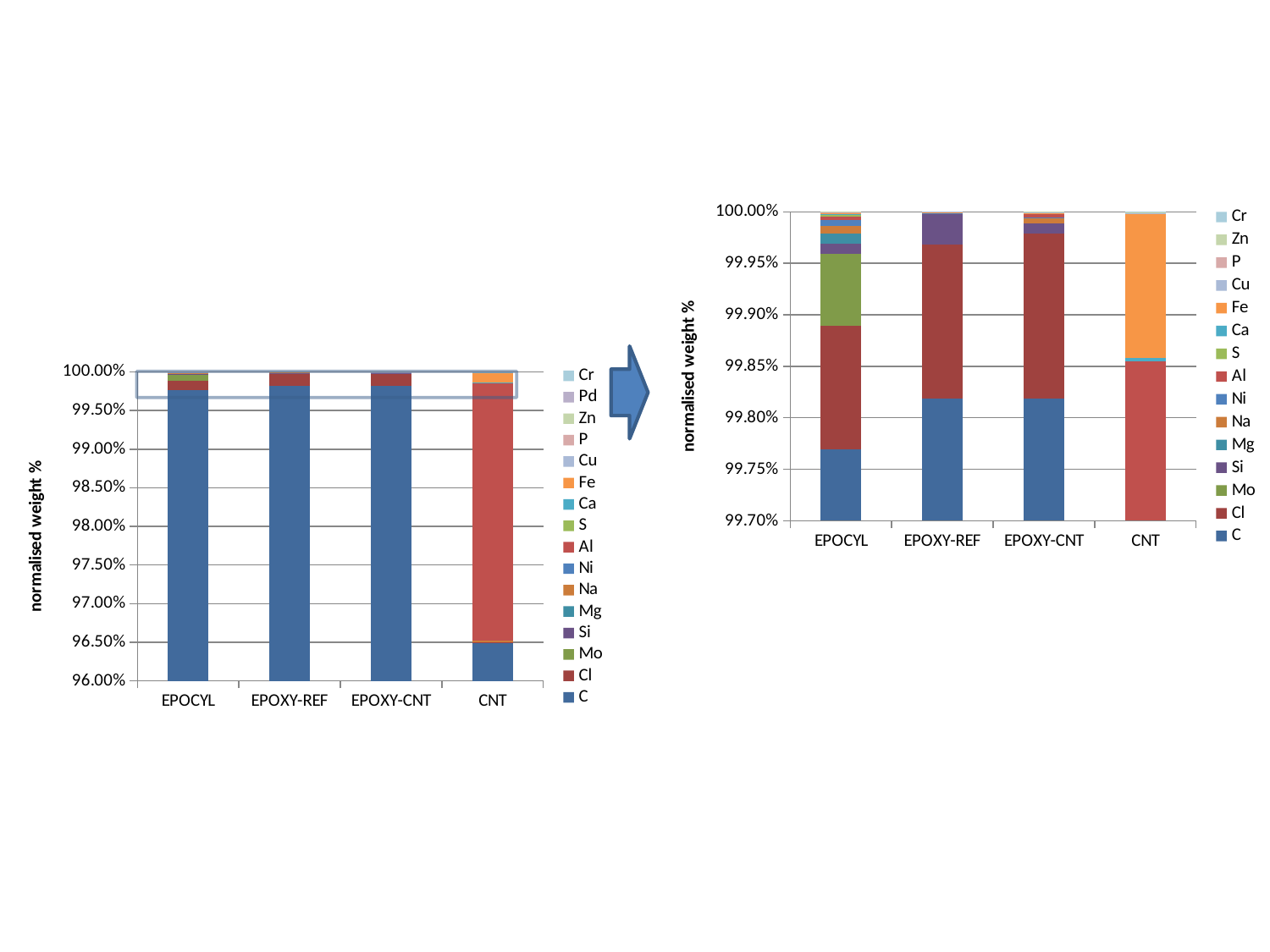

### Chart
| Category | C | Cl | Mo | Si | Mg | Na | Ni | Al | S | Ca | Fe | Cu | P | Zn | Cr |
|---|---|---|---|---|---|---|---|---|---|---|---|---|---|---|---|
| EPOCYL | 0.9976 | 0.0012 | 0.0007 | 0.0001 | 0.0001 | 7.5e-05 | 5.5e-05 | 3.5e-05 | 1.8e-05 | 1.1e-05 | 6e-06 | 4e-06 | 4e-06 | 2e-06 | 0.0 |
| EPOXY-REF | 0.99815 | 0.0015 | 0.0 | 0.0003 | 0.0 | 0.0 | 3e-06 | 0.0 | 0.0 | 0.0 | 6e-06 | 6e-06 | 0.0 | 0.0 | 0.0 |
| EPOXY-CNT | 0.99815 | 0.0016 | 0.0 | 0.0001 | 0.0 | 5.1e-05 | 3e-06 | 3.7e-05 | 0.0 | 0.0 | 1.2e-05 | 6e-06 | 0.0 | 2e-06 | 0.0 |
| CNT | 0.9649 | 5.9e-05 | 0.0 | 0.0 | 0.0 | 0.0002 | 4e-06 | 0.0333 | 0.0 | 3.1e-05 | 0.0014 | 4e-06 | 0.0 | 8e-06 | 8e-06 |
### Chart
| Category | C | Cl | Mo | Si | Mg | Na | Ni | Al | S | Ca | Fe | Cu | P | Zn | Pd | Cr |
|---|---|---|---|---|---|---|---|---|---|---|---|---|---|---|---|---|
| EPOCYL | 0.9976 | 0.0012 | 0.0007 | 0.0001 | 0.0001 | 7.5e-05 | 5.5e-05 | 3.5e-05 | 1.8e-05 | 1.1e-05 | 6e-06 | 4e-06 | 4e-06 | 2e-06 | 0.0 | 0.0 |
| EPOXY-REF | 0.99815 | 0.0015 | 0.0 | 0.0003 | 0.0 | 0.0 | 3e-06 | 0.0 | 0.0 | 0.0 | 6e-06 | 6e-06 | 0.0 | 0.0 | 0.0 | 0.0 |
| EPOXY-CNT | 0.99815 | 0.0016 | 0.0 | 0.0001 | 0.0 | 5.1e-05 | 3e-06 | 3.7e-05 | 0.0 | 0.0 | 1.2e-05 | 6e-06 | 0.0 | 2e-06 | 2.4e-05 | 0.0 |
| CNT | 0.9649 | 5.9e-05 | 0.0 | 0.0 | 0.0 | 0.0002 | 4e-06 | 0.0333 | 0.0 | 3.1e-05 | 0.0014 | 4e-06 | 0.0 | 8e-06 | 0.0 | 8e-06 |
